# Satellite data for environmental justice: a scoping review of the literature in the United States

**DOI:** 10.1088/1748-9326/ad1fa4

**Published:** 2024

**Authors:** Tanya Kreutzer Sayyed, Ufuoma Ovienmhada, Mitra Kashani, Karn Vohra, Gaige Hunter Kerr, Catherine O’Donnell, Maria H Harris, Laura Gladson, Andrea R Titus, Susana B Adamo, Kelvin C Fong, Emily M Gargulinski, Amber J Soja, Susan Anenberg, Yusuke Kuwayama

**Affiliations:** 1 School of Public Policy, University of Maryland, Baltimore County, Baltimore, MD, United States of America; 2 Department of Aeronautics and Astronautics, Massachusetts institute of Technology, Cambridge, MA, United States of America; 3 Environmental Public Health Tracking Program, Division of Environmental Health Science and Practice, National Center for Environmental Health, US Centers for Disease Control and Prevention, Atlanta, GA, United States of America; 4 Oak Ridge Institute for Science and Education, Oak Ridge, TN, United States of America; 5 Department of Geography, University College London, London, United Kingdom; 6 Milken Institute School of Public Health, George Washington University, Washington, DC, United States of America; 7 Environmental Defense Fund, New York, NY, United States of America; 8 Marron Institute of Urban Management, New York University, New York, NY, United States of America; 9 New York University Grossman School of Medicine, New York, NY, United States of America; 10 Center for International Earth Science Information Network, The Climate School, Columbia University, New York, NY, United States of America; 11 National Institute of Aerospace, Hampton, VA, United States of America; 12 NASA Langley Research Center, Hampton, VA, United States of America; 13 Author Kreutzer Sayyed, author Ovienmhada and author Kashani contributed equally to this work.

**Keywords:** environmental justice, satellite data, remote sensing, public health

## Abstract

In support of the environmental justice (EJ) movement, researchers, activists, and policymakers often use environmental data to document evidence of the unequal distribution of environmental burdens and benefits along lines of race, class, and other socioeconomic characteristics. Numerous limitations, such as spatial or temporal discontinuities, exist with commonly used data measurement techniques, which include ground monitoring and federal screening tools. Satellite data is well poised to address these gaps in EJ measurement and monitoring; however, little is known about how satellite data has advanced findings in EJ or can help to promote EJ through interventions. Thus, this scoping review aims to (1) explore trends in study design, topics, geographic scope, and satellite datasets used to research EJ, (2) synthesize findings from studies that use satellite data to characterize disparities and inequities across socio-demographic groups for various environmental categories, and (3) capture how satellite data are relevant to policy and real-world impact. Following PRISMA extension guidelines for scoping reviews, we retrieved 81 articles that applied satellite data for EJ research in the United States from 2000 to 2022. The majority of the studies leveraged the technical advantages of satellite data to identify socio-demographic disparities in exposure to environmental risk factors, such as air pollution, and access to environmental benefits, such as green space, at wider coverage and with greater precision than previously possible. These disparities in exposure and access are associated with health outcomes such as increased cardiovascular and respiratory diseases, mental illness, and mortality. Research using satellite data to illuminate EJ concerns can contribute to efforts to mitigate environmental inequalities and reduce health disparities. Satellite data for EJ research can therefore support targeted interventions or influence planning and policy changes, but significant work remains to facilitate the application of satellite data for policy and community impact.

## 1. Introduction

Numerous scholars have documented the consistency with which people from racial and ethnic minority groups, indigenous peoples, communities with indicators of lower socioeconomic status (SES), and other marginalized groups in the United States (U.S.) experience disproportionate exposure to environmental burdens, such as pollution, and unequal access to environmental benefits, such as green space [1, 2]. This pattern is often referred to as ‘environmental injustice’ and the movement and scholarship advocating for equity is called environmental justice (EJ) [3]. EJ is defined by the U.S. Environmental protection agency (EPA) as ‘the fair treatment and meaningful involvement of all people regardless of race, color, national origin, or income, with respect to the development, implementation, and enforcement of environmental laws, regulations, and policies’ [4]. In addition to the EPA, EJ has become an important social priority and policy goal at multiple levels of government.

Environmental data (e.g. measures of air pollution, heat, lead, and contaminants and pathogens in drinking water) are critical for efforts by the government and the public to characterize environmental injustice. Historically, these data have largely come from *in-situ* monitoring and computer modeling, often embedded within screening tools [5, 6]. For example, both fixed and mobile ground-based monitoring have been used to estimate the influence of traffic-related pollution on communities that are marginalized and minoritized [7, 8]. However, large gaps between stationary monitors preclude their ability to capture exposure hotspots and distinctions amongst demographic groups [9, 10], and mobile monitoring is often incomplete in time and space (e.g. covering weekdays or daytime only, over a limited number of weeks or years, or in only one neighborhood or city). While modeled datasets offer more spatial continuity than standalone ground monitoring data, they are often limited in spatial granularity and require ground-truthing [11, 12]. Screening tools that combine environmental and socioeconomic or demographic information in a mapping interface are also used to visualize communities that are environmentally burdened. While these tools have made data publicly available for characterizing environmental injustice, they are only as reliable as the environmental datasets on which they are based. The EPA’s online screening tool, EJscreen, the council on environmental quality’s climate and economic justice screening tool (CEJST), and other state-specific tools, such as the Maryland EJSCREEN Mapper, all use some environmental inputs that are out-of-date and at coarser spatial resolutions that may miss more current and localized environmental hazards [13–15]. In addition, other variables related to EJ concerns, such as extreme heat and past flood inundation, are not currently included in these specific tools. These limitations raise important questions about patterns of inequities that might be missed or misidentified.

Satellite Earth observations (EO) can help address some of the gaps in EJ measurement and monitoring, which in turn can enhance the rigor and social impact of EJ research. As defined by the Group on EO, ‘EO’ refers to information collected about our planet to assess the status of, and changes in, the natural and human-made environment [16]. EO includes *in-situ* measurements from field work, aerial photography, and space-based or remotely-sensed data. In this paper, we use the term ‘satellite data’ to refer to satellite EO, also commonly referred to as satellite remote sensing. Satellites detect measurements of radiation from different parts of the electromagnetic spectrum, or gravitational anomalies, to characterize Earth’s physical, chemical, and biological systems. The increased technical capability of satellite data can provide spatially complete global coverage at medium and high spatial and temporal resolutions. When merged with socioeconomic, demographic and health data, satellite data present an opportunity for EJ research to be conducted with greater breadth and depth than ever before. Specifically, satellite data provide increased capability for multi-temporal studies at the temporal revisit time of the satellite and enable study of small-area or neighborhood-level exposures and vulnerabilities.

Satellite data have been used to identify unequal access to green space and disparities in exposure and vulnerability to heat and air pollutants, among other environmental investigations [17–19]. While there is a growing body of literature at the intersection of satellite data and EJ, only a limited number of articles synthesize scholarship on this topic. In one review, Weigand *et al* [20] considered four environmental categories examined by existing studies (green space, air pollutants, noise, and heat), outlined how satellite data could be used for the derivation of each, and discussed nuances of integrating satellite data with differently-scaled socioeconomic data in EJ research as well as limitations of satellites for EJ analysis [20]. However, the paper did not use a transparent methodology to identify previous literature on the scientific findings about EJ enabled by satellite data and given the rapid pace of research in this area, is now outdated. Other articles have reviewed advances and findings on using satellite data for exploring neighborhood-scale air pollution inequities and health applications [21, 22], but only considered air pollution and did not report reproducible literature search methods. Additionally, only one of these articles [21] explicitly focused on EJ. These articles do not provide clear summaries of EJ-relevant satellite datasets and their sources, the provision of which could make satellite data more accessible for EJ practitioners. Lastly, they do not reflect on the potential value of satellite data to support policy development and planning initiatives which are aimed at remedying environmental injustices. There is a need for structured analysis that synthesizes the findings from literature using satellite data for a broader set of environmental hazards in EJ research, identifies relevant satellite datasets, and illustrates the potential value of satellite data for EJ impact.

The aims of this scoping review are to (1) explore trends in study types, topics, geographic scope, and satellite datasets used to research EJ, (2) synthesize findings from studies that use satellite data to characterize disparities and inequities across socioeconomic groups for various environmental categories, and (3) capture how satellite data are relevant to policy and real-world impact. A scoping review is the most appropriate method because this body of literature has not been previously reviewed in a comprehensive way or mapped into its key characteristics and emerging evidence [23]. We review articles in which authors combined satellite data with socioeconomic, demographic, and/or health data to explore environmental disparities. We synthesize findings regarding exposure or vulnerability to several environmental burdens and lack of access to environmental benefits that disproportionately impact certain groups along a variety of demographic and socioeconomic characteristics. Additionally, we tabulate relevant satellite data sources and tools used across the included studies. Finally, we highlight the strengths and limitations of satellite data for EJ as revealed through the synthesis and discuss implications through a public health and policy lens.

## 2. Methods

We conducted a scoping review of the literature using methodology that closely followed the 5-step framework outlined by Arksey and O’Malley [24] and the reporting guidelines outlined by the PRISMA extension for scoping reviews (PRISMA-ScR) [24, 25]. These steps included (1) identifying the research question (2), identifying relevant studies (3), selecting studies (4), extracting data from selected studies, and (5) summarizing, analyzing, and reporting results.

We addressed the following questions for this review: what are the publication trends in study design, environmental categories, geographic scope, and satellite datasets used in EJ studies? What EJ disparities and inequities^13^ have been identified in the peer-reviewed literature using satellite data and socioeconomic, sociodemographic, or public health data? How are satellite data applied in policy and intervention efforts to mitigate EJ inequities?

### 2.1. Search strategy

This review was a joint effort between scholars from the University of Maryland Baltimore County (UMBC) school of public policy and the national aeronautics and space administration’s (NASA) health and air quality applied science team’s satellite data for environmental justice (SD4EJ) tiger team. The literature search process consisted of two phases. In Phase 1^14^, conducted between September 2021 and December 2021, the UMBC team used keyword searches to identify literature related to the following broad environmental categories around which the scoping review was initially organized: (1) urban green space (UGS) (later changed to ‘green space’), (2) urban heat (later changed to ‘temperature’), and (3) air pollution. In this process, we used combinations related to the following search terms: environmental (in)justice, environmental (in)equality, satellite, satellite data, remote sensing, EO, race/racial, ethnic/ethnicity, inequality, inequity, green space, temperature, air pollution, pollution, flood, and health disparities. This initial search was conducted by manually exploring electronic databases via the Albin O. Kuhn Library at UMBC using keyword combinations summarized in table 1. Databases explored in the literature search included: EBSCO (such as academic search ultimate, science direct, and MEDLINE), clarivate web of science, google scholar, and JSTOR. PLOS one and researchgate were used to find two articles that were listed in other databases but were not available as full text. Reference lists from studies identified through the searches were hand-vetted by two UMBC reviewers to incorporate studies that may have been missed in the keyword searches. Articles returned via the keyword searches that were relevant to the guiding questions of this review but did not fit the initial environmental categories (i.e. green space, temperature, and air pollution) were retained, creating a fourth category of ‘other environmental hazards’.

In Phase 2 of the literature search, conducted between August 2022 and February 2023, the expanded SD4EJ Tiger Team identified additional studies by searching for literature from specific scholars known to our team and applying a second round of hand searches in google scholar using the keyword search strings.

### 2.2. Relevance screening and eligibility criteria

Relevance screening was applied to all studies identified in both Phase 1 and 2 searches. Initial relevance screening began with a review of titles and abstracts by a minimum of two reviewers. To guide our literature search and inclusion criteria, we used the EPA’s definition of ‘EJ’, which reflects how patterns of environmental injustice are often found along lines of race, ethnicity, and SES in the U.S [2, 13, 26]. To include only a body of literature consistent with the EPA’s definition, we only considered studies based in the U.S. Multi-city international studies that included U.S. cities in their analysis were similarly excluded. Other criteria applied at this stage were that the article be peer-reviewed^15^ and published in english between 2000–2022.

Articles that were not excluded based on these criteria at the title and abstract screening stage were considered in the full-text review stage. A minimum of two authors applied inclusion criteria to manuscripts at this step and agreed on a decision and reasons for inclusion or exclusion. When a consensus for an article could not be reached by a subset of authors, all authors met to deliberate on a decision.

During the full-text review stage, inclusion was further evaluated based on the source of the environmental datasets used in the article. We carefully assessed these environmental datasets, which in some cases required reading additional articles or metadata. Studies were classified into two broad categories of inclusion based on their treatment of satellite data: ‘direct satellite measurements’ and ‘indirect satellite measurements’. When only quality assurance measures, data cleaning, or basic statistical techniques were applied to remotely sensed imagery from satellites, we considered these measurements to be ‘direct’ satellite measurements. When research requires estimates of surface-level quantities (rather than columnar densities), or when interference from atmospheric diffusion and absorption complicates direct use of satellite retrievals, satellite data can be combined with other datasets and tools (e.g. models) to increase usability. In such cases, we considered these hybrid datasets to be ‘indirect’ satellite measurements. To be included in this review, the articles’ authors must have made an explicit connection to socioeconomic or demographic disparities, outcomes, or inequities in a way that used direct or indirect satellite-derived data as an independent variable. Studies that solely used geographic information systems or mapping tools in their methodology without the use of satellite-derived data as an independent variable were excluded.

### 2.3. Data analysis and synthesis

Included articles were grouped into primary and secondary environmental categories (i.e. green space, temperature, air pollution, and other environmental hazards). Primary and secondary environmental categories were determined based on the independent variable that the satellite instrument measured in relation to EJ. For example, a study that examined urban heat islands (UHIs) and the modifying effect of air pollution but only used satellite instruments to measure temperature would be primarily categorized as a ‘temperature’ study and secondarily as an ‘air pollution’ study.

The following 14 attributes were extracted and recorded in a spreadsheet for the remaining studies: author(s), year, satellite instrument(s) or product(s) used, spatial resolution of instrument or data product(s), primary and secondary environmental category, environmental variable(s) measured, study location, geographic scope (e.g. city, county, state, region, multi-city, or national), study observation design (e.g. cross-sectional, multi-temporal^16^, or both), study type (e.g. differential exposure, differential vulnerability, etc), spatial scale of analysis (e.g. census tract, city, block group), social categories of analysis (e.g. race, ethnicity, income), key findings, and other non-environmental datasets used (e.g. American community survey census data). The extracted attributes and their explanations can be found in table 1 of the supplementary file. Extracted data are further summarized and tabulated into three tables, which include (1) the total number of articles categorized across all extracted attributes (see table 2 of the supplementary file), (2) summaries of the satellite and satellite-derived datasets used, their availability, and their sources (see table 3 of the supplementary file), and (3) summaries of the socioeconomic datasets (e.g. U.S. Census data) used in the included literature (see table 4 of the supplementary file). Although not extracted into the spreadsheet, we also noted and synthesized insights from studies which discussed the potential for satellite data to be used to advance EJ through government regulations, programs, and guidance, or city and community planning.

We provide narrative accounts of the studies arranged thematically by environmental category into four subsections: green space, temperature, air pollution, and other environmental hazards. We do not assess the quality or risk of bias in the included studies.

### 2.4. Study limitations

Our retrieved literature may have been limited due to unintentional omission of terminology used in EJ-relevant literature across different fields. In addition, as our inclusion criteria required studies be peer-reviewed, exclusively U.S. based, and published by 31 December 2022, our synthesis, discussion, and conclusions may miss other important trends or insights on satellite data for EJ, such as those published in the gray literature, internationally, or outside our specified date range.

## 3. Results

We present the descriptive results from our scoping review and then the results of our synthesis in four subsections corresponding to the environmental categories of green space, temperature, air pollution, and other environmental hazards. We also present a results subsection that describes how authors used satellite data to suggest or endorse policy changes and other interventions.

### 3.1. Characteristics of the included studies

**Figure 1.**
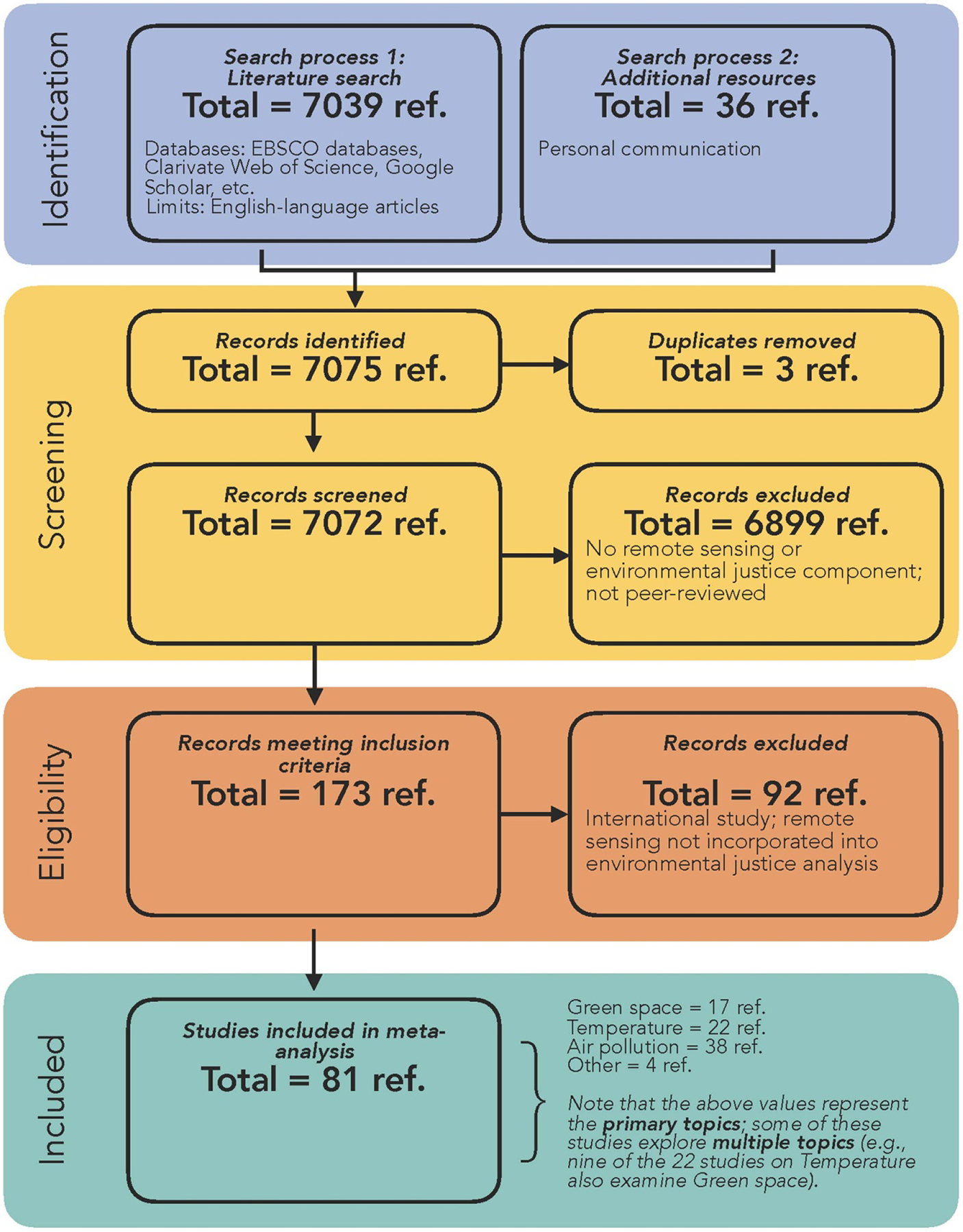
Summary of study identification and selection following the preferred reporting items for systematic reviews and meta-analyses (PRISMA) guidelines.

The literature search, which included database searches and literature retrieved via personal communication and hand searches, returned 7075 articles of which 7072 unique articles underwent title and abstract screening. Among these, 173 studies underwent full-text screening (see figure 1). After screening, 81 articles met all inclusion criteria and underwent full data extraction and analyses (see tables 2(A)–(D) for the complete list of included articles with select attributes shown).

**Figure 2.**
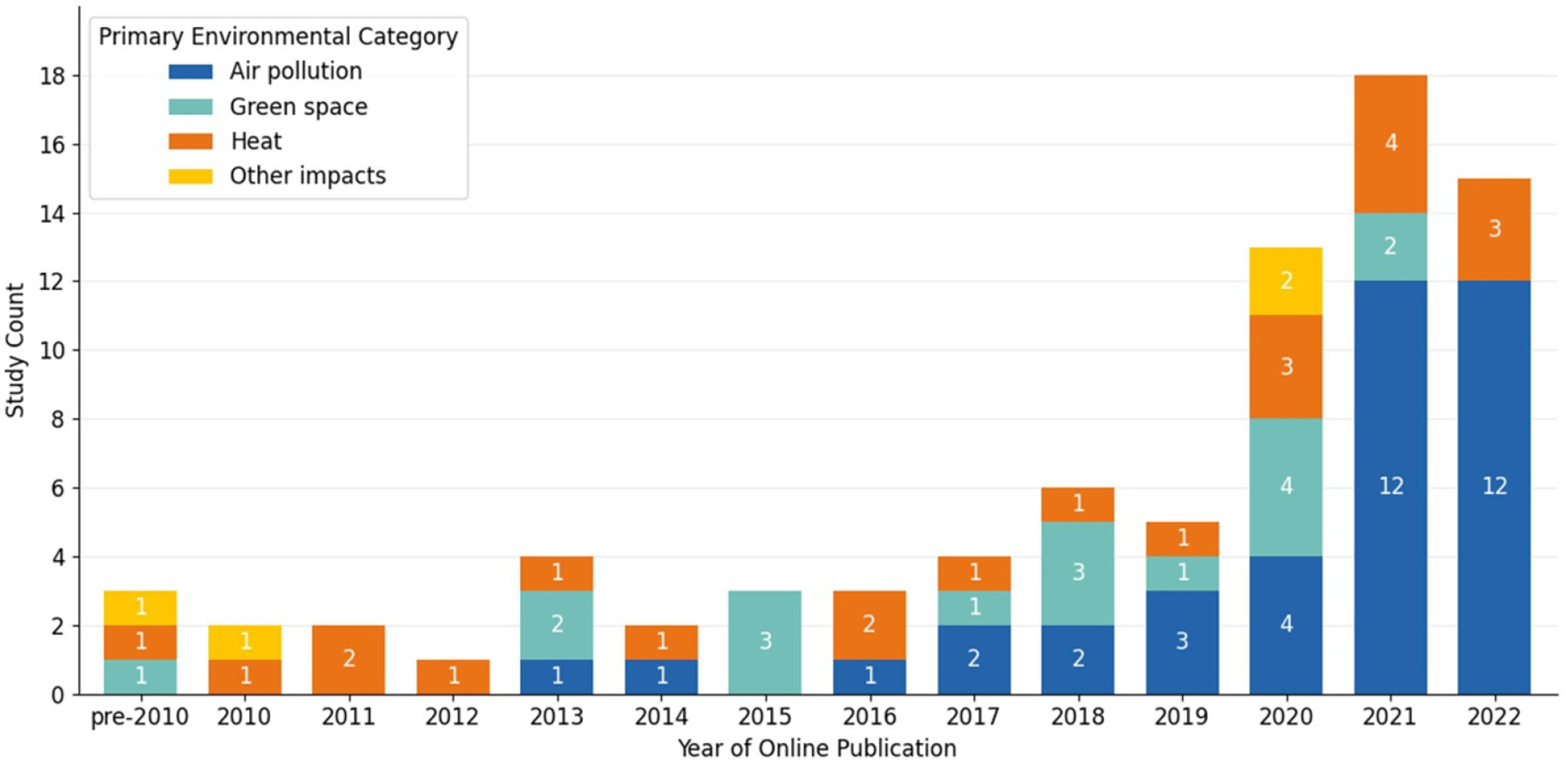
Number of studies included in our review (*n* = 81) by publication year and primary environmental category of interest. In the pre-2010 bin, there was one study each in 2005 (other environmental hazards) [110], 2007 (temperature) [55] and 2009 (green space) [40].

**Figure 3.**
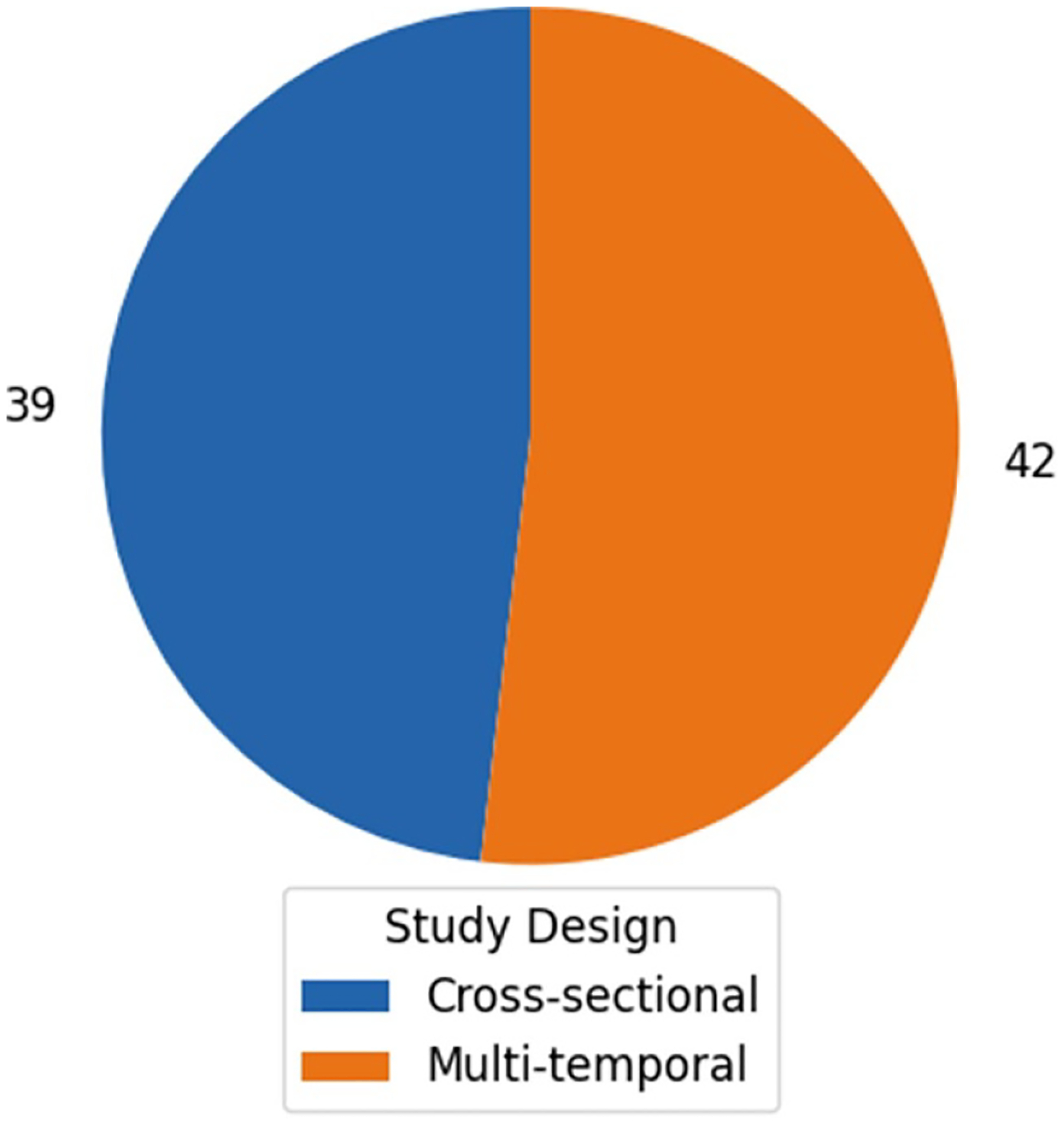
Breakdown of total studies included in our review (*n* = 81) by study design.

**Figure 4.**
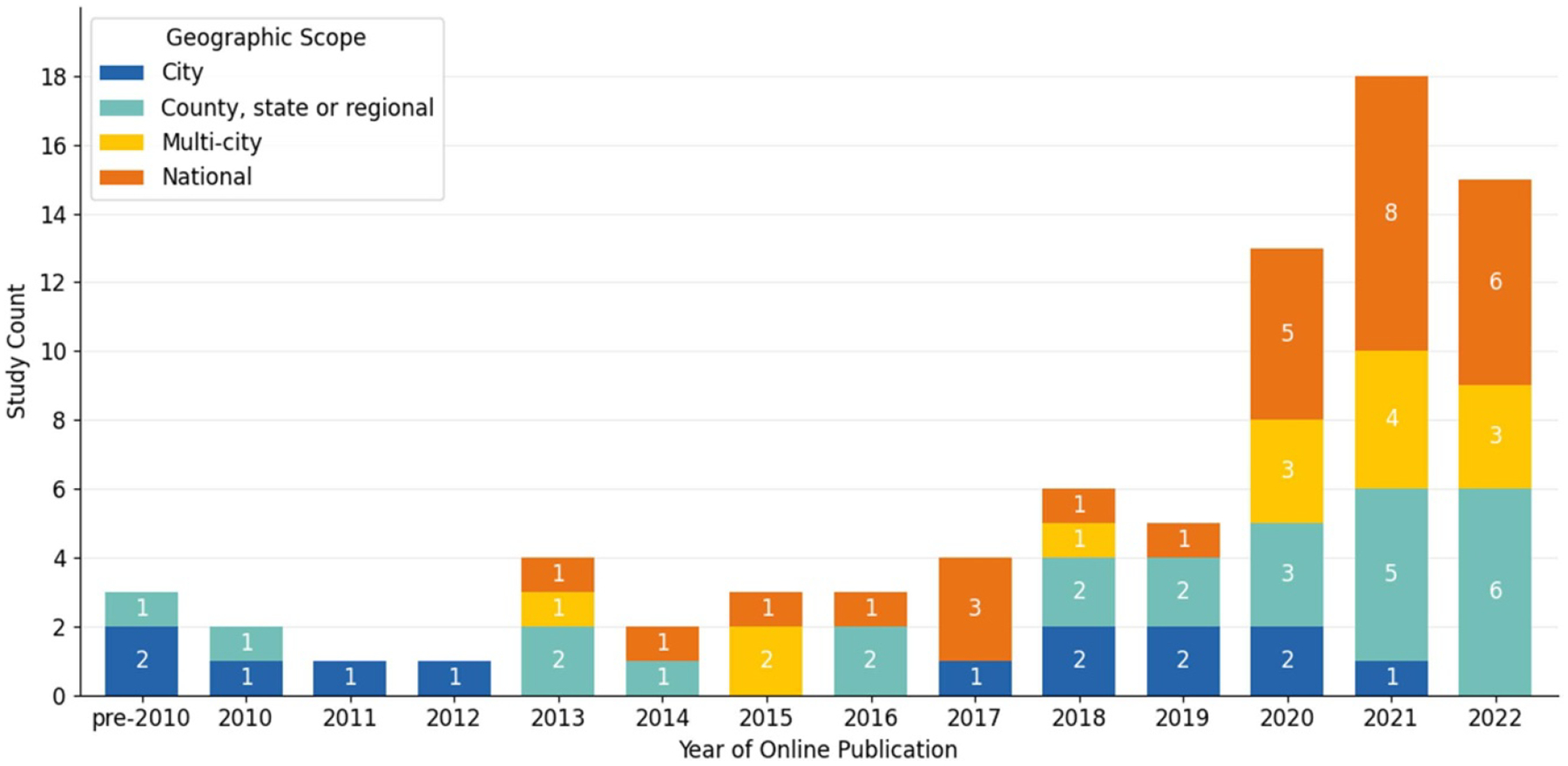
Number of studies included in our review (*n* = 81) by publication year and geographic scope. In the pre-2010 bin, a 2005 study [110] had a state-wide geographic scope, and a 2007 [55] and 2009 [40] study had a city-wide geographic scope. The category ‘multi-city’ is taken to describe generally urbanized areas as some authors compare counties alongside cities in multi-city studies [52, 54, 67, 103, 105].

**Figure 5.**
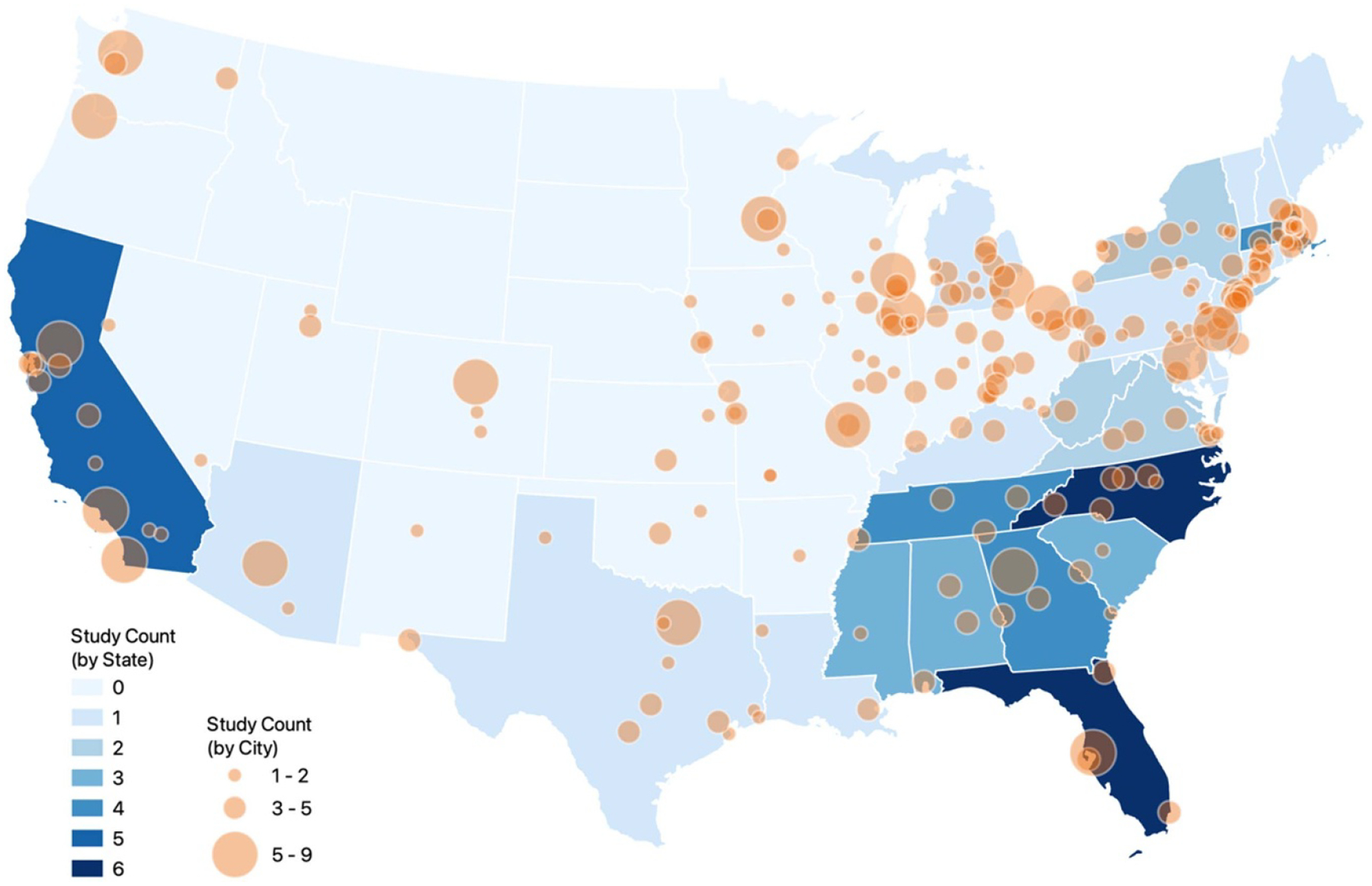
Map of studies included in our review by study location. The shaded value ‘study count (by state)’ is the number of studies that include that entire state or a county in that state in their analysis (*n* = 24/81). The bubble size ‘study count (by city)’ corresponds to the number of studies conducted at the city or multi-city level, where ‘city’ is taken to also include generally urbanized areas (*n* = 27/81). One multi-city study [53] could not be mapped due to data unavailability. 29 studies with a ‘national’ geographic scope conducted at various geographic scales (i.e. census tract, census block group, zip code etc.) are not mapped in this figure.

The 81 unique articles were published between 2005 and 2022 with more than half (*n* = 46) published between 2020 and 2022 (see figure 2). In terms of study design, there was a near-even distribution of multi-temporal (*n* = 42) and cross-sectional (*n* = 39) studies (see figure 3). Studies ranged in geographic scope: 29 were national; 24 were at the regional, state, or county level; and 28 were at the city or multi-city level (see figures 4 and 5). Race (*n* = 66) and ethnicity (*n* = 46) were the most common social constructs over which injustice was examined.

The included studies leveraged satellite data products to explore inequities in air pollution (*n* = 38), green space (*n* = 17), temperature (*n* = 22), or other environmental hazards (*n* = 4) such as flooding, light pollution, land-use, and gas flaring (see figure 2). Eleven out of the 81 studies concurrently investigated more than one environmental hazard, such as temperature and green space (*n* = 9), temperature and air pollution (*n* = 1) and green space and air pollution (*n* = 1). Table 3 describes the most commonly used satellite instruments (used by three or more studies), grouped by environmental category. The full list of satellite instruments used as well as their technical specifications are in table 3 of supplementary materials.

### 3.2. Green space

Disparate distribution of green space is, and continues to be, shaped by historical and contemporary systemic racial and ethnic discrimination and segregation [27, 28]. The EPA defines green space as land that is partly or completely covered with grass, trees, shrubs, or other vegetation, including parks and community gardens [29]. Previous studies have linked green space to improved physical, social, and mental health outcomes, a heightened sense of overall well-being [30–36], reductions in morbidity and mortality risks [37], mitigation of urban heat [38], and reduced air and noise pollution [39]. Lack of access to green space is widely recognized as a form of environmental injustice. Racial and ethnic minority residents in communities with lower income and limited access to green space may experience worse overall health outcomes and a lower quality of life than non-minority, wealthier residents in areas with higher access to green space [35].

Satellite data can reveal information about vegetation density, leaf area index, vitality and health, and ecosystem type. Commonly used indicators include normalized differential vegetation index (NDVI) and the enhanced vegetation index (EVI) [114, 115]. Vegetation indices, including NDVI and EVI, indicate the relative greenness or health of vegetation based on the contrast between the maximum reflection in the near infrared band caused by leaf cellular structure, and the maximum absorption in the red band due to chlorophyll pigments. Satellite data can also be used in true color red-green-blue (RGB) to visually assess vegetative features. The vegetation indices and other direct and derived satellite products aid in characterizing green space through variables such as UGS, urban tree canopy cover (UTC), and tree canopy cover (TCC).

We identified 17 articles that used direct or indirect satellite data to analyze differential exposure, and in some cases differential vulnerability, to UGS, UTC, and TCC. While most studies use RGB imagery or NDVI as a direct satellite product [43, 45, 46, 48–51, 53, 54, 116] several studies used indirect satellite-derived products, such as the national land cover datasets (NLCD) and the national agricultural imagery program, to explore population disparities in green space exposure [17, 41, 42, 44, 52].

#### 3.2.1. Distribution and accessibility of green space

Five studies used direct and indirect measurements of urban greenness from satellite data (NDVI and TCC) to show that nationally, communities with larger proportions of people who are racial and ethnic minorities and with increased degrees of racial segregation, had less presence of urban greenness [17, 43, 45, 52, 54]. One multi-temporal study leveraged satellite data to show that these disparities persisted in census tracts nationally over a 10 year period from 2001 to 2011 [45]. The same finding of inequitable distribution of greenness among racial/ethnic minority groups was also found in two studies that considered smaller geographic areas on the scale of individual cities and states [40, 41]. The only study to consider distribution of green space for immigrant communities, as measured by the designation of ‘foreign-born’ from the U.S. Census Bureau, found that these communities also had less greenness [51]. Other studies considered economic indicators in communities; for example, Schwarz *et al* [44] and Saporito and Casey [43] found that urban greenness correlates with higher incomes and lower degrees of economic segregation.

Two studies used satellite data and indicators of proximity to evaluate accessibility to parks and the quality or features of parks available to different racial/ethnic groups. The studies did not find significant evidence of a disparity in park accessibility in Illinois or Phoenix, AZ, metro area census block groups [41, 47]. However, one found evidence that there were variations in the quality and features of parks for different ethnic groups [47]. Specifically, they found that Latinx residents living in Phoenix had more access to trees whereas non-Latinx residents had more access to diverse natural features and open space.

In one study, authors leveraged the multitemporal advantage of satellite data to evaluate the suitability and potential biases of urban greening indicators for EJ research over time. Schwarz *et al* [48] compared Landsat NDVI data from three different years (1980, 2000, 2014) to census tract information on housing vacancy. In their study, they showed that high vacancy rates were strongly correlated with indicators of populations that are minoritized or are experiencing poverty. Due to this association, they said, ‘under prevailing EJ theories regarding urban greening, we would expect that NDVI values would be lower in high-vacancy tracts where populations that are underserved are concentrated’. They found that low vacancy was associated with higher NDVI values in 1980 and 2000, but by 2014 there was no association between NDVI and housing vacancy. The authors speculated that spontaneous and non-amenity vegetation, such as overgrowth, in neighborhoods with high-vacancy may be biasing the results towards the null. This study showcases the ability for satellite data to identify limitations in current accepted methodologies and theories in EJ research.

In combination with other data, satellite data may be useful for aligning EJ efforts with redistributive policy measures. Multiple studies reflected on how satellite data could inform evidence-based planning and interventions targeted at equitable distribution to improve access to environmental benefits in areas with populations that are vulnerable. For example, authors of several articles described how their findings could be used to improve availability and accessibility to green space [40, 45, 49, 52, 53], and increase UTC and UGS [50, 57]. One study used satellite data to estimate a reduction in premature deaths based on projections from Philadelphia’s Greenworks Philadelphia plan to increase UTC in neighborhoods that are low-income and neighborhoods with higher proportions of residents of color [50]. The authors estimated that the expansion of UTC by 30% was associated with an estimated reduction of 403 premature deaths annually overall, including 244 premature deaths in areas of lower SES. Another study assessed the adequacy of Detroit’s future city plan which includes heat mitigation efforts through greening [65]. They found that the populations most vulnerable to heat in Detroit, namely Black populations, would have the highest percent access to green space within the area of the proposed plan.

#### 3.2.2. Health as a function of green space

Six articles evaluated satellite measurements of green space as a factor of differential vulnerability or as an inclusive factor in a health impact analysis [42, 46, 49, 50, 53, 116].

A national study showed that a higher ratio or presence of green space was associated with lower racial disparities in COVID infection rates [53]. Another study estimated that increasing UTC in the city of Philadelphia would prevent a higher proportion of premature deaths in areas of lower SES [50]. Other studies have found that the presence of more green space was associated with reduced odds of Alzheimer’s disease and depression in neighborhoods with lower SES, reduced risk for hospitalizations associated with particulate matter of 10 *μ*ms or less (PM10), and a reduced odds of heat-related cardiovascular mortality in elderly populations [42, 46, 116]. In contrast, a study conducted by Son *et al* [49] concluded that residential greenness did not lead to significant differences in health disparities attributable to air pollution.

### 3.3. Temperature

In many parts of the United States, extreme temperatures -both heat and cold- pose substantial health risks, such as asthma exacerbation and heat-related illness, which disproportionately burden populations that are marginalized [121, 122]. People who live in cities are especially at risk of extreme heat due to the UHI effect, which is the ability of built infrastructure, such as buildings and roads, to absorb the sun’s heat more readily than natural landscapes [71]. Exposure to extreme heat from UHI and anthropogenic climate change is responsible for rising heat-related morbidity and mortality rates in urban settlements globally and is the leading weather-related cause of death in the U.S [123, 124].

While satellites cannot directly measure air temperature, which is more closely related to health effects [125], satellites can directly detect land surface temperature (LST) by measuring reflected light from the infrared portion of the electromagnetic spectrum. There are various ways to compute LST, including combining satellite data with additional meteorological and land-use parameters. For example, the method endorsed by the United States geological survey (USGS) uses NDVI, the proportion of vegetation, and emissivity as input variables [126]. The surface UHI effect is then computed as the difference in LST between urban and non-urban areas. LST is often measured alongside other satellite image indices such as the normalized difference built up index or used to calculate cumulative risk indices. Satellites, such as those from the NASA and USGS landsat program, provide 30-meter spatial resolution enabling analysis of heat inequities with more continuity than possible with ground monitors. Of the 22 articles in this review with a focus on heat, 18 articles use LST or other indices to assess urban heat, heat risk or vulnerability [18, 55–61, 64–69, 71–74] and four articles use modeled air temperature derived from satellite measurements of surface temperature [62, 63, 70, 75].

#### 3.3.1. Heat exposure

Using satellite data to characterize heat exposure is challenging due to the aforementioned limitation that satellites do not directly measure air temperature, which is more relevant to human health than is LST [125]. However, satellite data still provide insight on the contiguous spatial patterns and differences in heat exposure within populated areas, which ground-based temperature measurements cannot provide. Nineteen articles used satellite data to characterize urban heat exposure for different population groups across geographies in the United States. Sixteen articles found that communities with lower SES and other measures of social vulnerability experienced greater urban heat vulnerability [59] and exposure [18, 55–58, 61, 64, 66–72, 75].

The 19 articles had various findings on exposures for minority racial/ethnic groups in different geographies. In thirteen studies, Black, Hispanic and Asian populations had greater exposures to heat in the majority of investigated areas [18, 55, 58, 61, 65, 66, 68, 69, 72, 73, 75]. One national study showed that statistically significant racial heat disparities persisted when adjusting for income and when restricting the analysis to smaller rural areas, which are often excluded from heat studies [72]. Five studies found that measures of segregation partially explain these thermal inequities for different racial and ethnic groups [18, 61, 67, 68, 75]. In contrast, one study in Atlanta found that populations that are historically marginalized (predominantly Black persons with less access to education and wealth) had less exposure to high temperatures, potentially driven by more extensive UTC and less impervious surfaces [74]. Two articles used satellite data and other data sources to observe built environment features and how they contributed to urban heat disparities. A national study found that systematically, the disproportionate heat exposure faced by racial and ethnic minority communities was due to more built-up neighborhoods, less vegetation, and—to a lesser extent—higher population density [72]. A Philadelphia study found that presence of vacant land and impervious surfaces has a stronger relationship with high temperatures than does NDVI [64], the latter having been previously connected with temperature variations via vegetation’s cooling effect [127, 128].

Authors of several articles described how their findings could be used to improve access to cooling resources for heat mitigation [58, 60, 64, 66]. For example, a study in Atlanta found that by using satellite data they could confirm that the majority of the city’s climate resilience planning is appropriately focused on the areas with the highest heat exposure [74]. In some studies, satellite data were useful for identifying locations which could benefit from expanded monitoring stations [62, 63].

#### 3.3.2. Temperature and mortality

Three multi-temporal studies leveraged the high spatial resolution of satellite data to estimate temperature-related mortality for different population groups. Harlan *et al* [60] identified that neighborhood scores on surface temperature, socioeconomic vulnerability and elderly/isolation were best fit at predicting odds of deaths from heat exposure in Arizona. One study found that Black populations had significantly higher associations of mortality with increases in temperature [62], while another found smaller relationships between heat and mortality for different racial groups, with Black populations more vulnerable to changes in mean temperature than White populations [63]. Both studies found that people in less urban areas were more sensitive to increases in temperature. They also observed that satellite data improved temperature estimates in rural areas that have more sparse weather monitoring stations, and enabled Shi *et al* [63] to conduct a study that controlled for fine particulate matter of 2.5 *μ*ms or less in diameter (PM_2.5_), which can bias results on health effects of temperature [129].

### 3.4. Air pollution

Air pollution exposure is associated with a broad range of health outcomes, including asthma, respiratory infections, cardiopulmonary disease, lung cancer, adverse birth outcomes, and cognitive disorders [1, 89, 130]. Some air pollutants including nitrogen dioxide (NO_2_), ozone (O_3_), sulfur dioxide (SO_2_), and carbon monoxide (CO) are observable by satellite instruments through retrieval algorithms (‘direct satellite measurements’; see methods). Models incorporating satellite measurements can also be used to estimate ground level concentrations of these pollutants (‘indirect satellite measurements’). Other species, such as PM_2.5_, can be inferred by combining direct measurements of aerosol optical depth (AOD), which is a measure of light attenuation by atmospheric aerosols [131], with physical or statistical models.

Of the 38 studies in this review with a focus on air pollution and EJ, seven incorporated direct measurements of NO_2_ and AOD, while 31 studies used indirect satellite measurements of PM_2.5_, PM_10_, O_3_, NO_2_, SO_2_, CO, and NO_X_. Among the studies that used indirect satellite measurements, the most common focus was surface-level PM_2.5_ derived from a chemical transport model that related direct measurements of AOD from multiple satellite instruments to near-surface PM_2.5_ concentrations [118, 132]. Other studies that used indirect satellite measurements relied on statistical models, chemical transport models, or machine-learning models that incorporated satellite measurements of air pollution, land cover and land use data, meteorological data, and surface-level measurements of pollutants [94, 106, 119]. Despite the incorporation of geophysical or statistical models to generate these indirect satellite measurements, analyses of their performance against *in-situ* monitors found good agreement [133–136]. Among the 38 studies included in this review, 21 focused on PM_2.5_ as the primary pollutant of interest, while 8 focused on NO_2_, 8 examined multiple pollutants, and 1 focused on NO_x_.

#### 3.4.1. Air pollution exposure

Several multi-temporal studies used indirect satellite measurements to quantify inequities over multiple decades in pollutant concentrations across the U.S [19, 84, 94, 104] and within individual states [80, 108], while others focused on a single point in time [83, 92, 100, 106]. Across geographies, these studies reported substantial disparities in air pollution concentrations across racial/ethnic groups and economic strata [19, 80, 84, 92, 94, 104, 108], with racial/ethnic disparities typically larger and more consistent than disparities by income levels [19, 80, 94]. The observed degree of disparity also varied across pollutants, with NO_2_ generally displaying larger disparities than PM_2.5_ or other pollutants [80, 94]. Longitudinal studies consistently reported that while *absolute* disparities in pollution exposure have generally decreased over time as pollution concentrations have declined, *relative* disparities decreased to a lesser degree [19, 84, 94], or even increased [80, 104].

Recent research has also leveraged direct satellite measurements to characterize relative disparities in NO_2_ and PM_2.5_ exposures. Studies utilizing the TROPOspheric monitoring instrument (TROPOMI) to examine NO_2_ disparities in major urban centers in the U.S. reported lower NO_2_ concentrations in census tracts with greater proportions of White and higher income residents [86, 93, 101]. Several of these studies identified heavy-duty diesel traffic as a major contributor to intra-urban NO_2_ disparities. For example, across 52 urban centers evaluated by Demetillo *et al* [86], an estimated 60% drop in heavy-duty truck traffic on weekends was associated with a 40% reduction in the degree of exposure inequality, while Kerr *et al* [93] presented evidence that NO_2_ disparities that persisted throughout the 2020 COVID-19 lockdowns were in part due to continued heavy-duty diesel activity throughout that period. Other researchers used direct satellite measurements to examine the impact of COVID-19 shutdowns on racial/ethnic disparities in PM_2.5_ (using AOD as a proxy) in New York state [137] and NO_2_ in California [98].

Hrycyna *et al* explored the influence of historical discriminatory redlining on current pollution levels using direct satellite measurements from TROPOMI [103]. They reported that residents living in historically redlined neighborhoods were exposed to substantially higher NO_2,_ which is consistent with results reported by researchers examining associations between redlining and NO_2_ and PM_2.5_ pollution concentrations using indirect satellite measurements [105]. Other research leveraging indirect satellite measurements examined the influence of income inequality [78] and public housing development siting [100] on observed racial/ethnic and socioeconomic disparities in air pollution exposure.

#### 3.4.2. Air pollution health outcomes

Multiple national studies integrating administrative health data and satellite air pollution data found that vulnerability to the health effects of air pollutants is most pronounced among individuals and communities with lower SES [76, 79, 88, 97, 99, 109]. Several studies also found that Black and male individuals might experience the most pronounced health effects due to air pollutant exposure [79, 82, 87], though findings were not universal [10, 81, 95, 102] and might vary by pollutant [97]. Studies examined in this review incorporated a range of data sources, such as products from the U.S. Census Bureau (e.g. American community survey, decennial census) for population demographic and economic characteristics, and sometimes integrated fine-scale meteorological data [87, 96] and satellite-derived measures of greenness [96]. Several studies examined how health effects of air pollutants differed according to composite indices of area-level vulnerability, including the social deprivation index [88] or the area deprivation index [109]. For example, Wei *et al* [109] integrated spatially granular (1 km × 1 km) data on multiple air pollutants (PM_2.5_, NO_2_, O_3_) with national inpatient fee-for-service Medicaid claims data and ZIP code-level information on the area deprivation index. For all pollutants, associations with asthma hospitalizations were most pronounced among individuals living in areas with higher deprivation.

Using highly spatially resolved satellite data has also facilitated comparisons by urbanicity or across areas with varying levels of distance to the closest ground-based air quality monitor. For example, Kloog *et al* found that associations between long-term PM_2.5_ exposure and mortality were higher among individuals living closest to ground monitors (within 20 km) in Massachusetts, compared to individuals living farther away [76]. Other analyses found that associations between PM_2.5_ exposure and mortality among Medicare beneficiaries in North Carolina and Michigan were higher in rural areas compared to urban areas [96], whereas associations between NO_2_ exposure and mortality among Medicare beneficiaries in the Southeast U.S. were more pronounced in urban areas than in rural areas [95].

#### 3.4.3. Air pollution burden assessment and policy evaluation

In addition to exposure assessment and epidemiological studies on the health effects of air pollutants, satellite data have also been used to quantify differential burdens of air pollution in particular regions [89, 107] and inform evaluations of the impacts of air pollution policies on exposure disparities [77, 85, 138]. For example, Clark *et al* used indirect satellite measurements to estimate the potential health benefits of eliminating national racial/ethnic and socioeconomic disparities in NO_2_ exposure and proposed an environmental inequality ranking system by urban area, county, and state [77]. Several studies used satellite data to evaluate existing air pollution control policy or described how it could help inform future policy development or evidence-based planning towards alleviation of environmental hazards in areas with populations that are vulnerable. Currie *et al* used multitemporal satellite data to show the efficacy of the clean air act in reducing racial inequities in air pollution exposure through spatially targeted air quality regulations [85, 138]. Problems with widespread use of sparsely and unevenly sited regulatory monitors to measure compliance with the clean air act have been brought to light with satellite data. Sullivan and Krupnick [9] and Fowlie *et al* [139] showed how monitors cannot fully characterize nationwide PM2.5 exposure disparities or capture attainment versus non-attainment designations under the national ambient air quality standards of the clean air act, which could have enormous public health, welfare, and economic consequences if areas are inadvertently considered to be in attainment of these standards due to the use of monitors. Authors of other studies suggested that exposure to traffic-related air pollution could be partially mitigated by reducing emissions from the heavy-duty trucking sector through, for example, truck electrification and vehicle bans [86, 90, 93] and other city planning decisions. Satellite data also helped identify locations that could benefit from expanded *in-situ* monitoring of air pollution [140].

### 3.5. Other environmental hazards

We categorized four articles as examining other EJ hazards: artificial nighttime light, flaring from unconventional oil and gas development, mountaintop removal coal mining, and flooding.

While artificial nighttime light can have many benefits such as enabling nighttime economic and social activity, a growing body of research is linking artificial nighttime light to potential negative public health impacts, such as metabolic disorders and cancer [141]. Satellite sensors, such as NASA’s visible infrared imaging radiometer suite (VIIRS), can detect nighttime light in the visible part of the electromagnetic spectrum. This information is used in fusion datasets such as the Atlas of artificial night sky brightness. Using the atlas, Nadybal *et al* found that Black, Asian, and Hispanic people and Native Hawaiian/Pacific Islanders experienced considerably higher population-weighted mean exposures to nighttime artificial light than non-Hispanic White persons [113].

Gas flaring releases hazardous air pollution in the form of particulate matter and volatile organic compounds and contributes to light, noise, water and noxious odor pollution [112]. The VIIRS nightfire (VNF) satellite product can detect combustion sources such as flaring [142, 143]. Using the VNF product in a longitudinal study, Johnston *et al* [112] found that Latinx populations were exposed to twice as many nightly flare events within proximity to their homes than non-Hispanic Whites.

Mountaintop removal coal mining (MTM) is a form of surface coal mining associated with a variety of detrimental environmental effects, such as water pollution, flooding, and forest loss, and adverse health effects, such as heart disease, cancer, and birth defects [144, 145]. Satellite data can be used to identify MTM through visual indicators [146]. Evaluating Appalachian and non-Appalachian counties across four southern states, Hendryx used satellite-derived MTM location data and found that Appalachian communities with higher rates of poverty were disproportionately exposed to environmental health risks associated with MTM [111].

Flooding can cause immediate health impacts such as drowning, injuries, hypothermia, respiratory and lung diseases, and diseases from animals breeding in stagnant water [147]. Flooding can also have a long-term economic and social impact on livelihoods [148, 149]. Flooding can be identified from satellite imagery through both optical and synthetic-aperture radar (SAR) based methods [150, 151]. Focused on eastern North Carolina, Guidry and Margolis used flood maps derived from SAR satellite data and found that schools that were attended by majority African-American students and students with lower income are at a greater risk of being flooded [110].

## 4. Discussion

In alignment with other EJ literature [2], the majority of the reviewed 81 articles applying satellite data for EJ found evidence of unequal exposure to environmental burdens, including air pollution, heat, and other impacts, and unequal access to environmental benefits such as green space, along lines of income, race, ethnicity, and other socioeconomic and demographic markers. In general, people residing in lower-income neighborhoods or households and people who belong to racial and ethnic minority groups routinely had the largest inequities. That satellite-derived data reinforce conclusions established using other data sources underscores the persistence of environmental injustice in the U.S. The review also offers more novel synthesis on trends in the application space of satellite data for EJ, the technical advantages of satellite data for describing disparities and health impacts at spatial and temporal scales not previously possible, and the relevance of satellite data for use in policy contexts and other efforts to remedy injustice. We expand on each of these more novel contributions in the following subsections.

### 4.1. Trends in the application of satellite data for EJ

Looking across the studies, we found some key trends that can help guide future research. As shown in figure 2, most of the literature employed satellite data for air pollution research, with the environmental categories of green space and temperature represented at approximately half of the proportion of air pollution studies. Over time, the literature veered away from study sites at the level of the city and leveraged the spatial coverage of satellite data to do more multi-city and national research (see figure 4). Yet, we see that at the sub-national level, parts of the Pacific Northwest, Southwest, and Midwest may be underrepresented (figure 5). Existing studies are also mostly focused on urban spaces, and EJ concerns relating primarily to rural or agricultural contexts, such as land conservation and mining, are understudied using satellite data. Study design (figure 3) is nearly evenly split between cross-sectional and multi-temporal. Some of this trend may owe to data availability given that studies conducted at earlier time periods within the scope of our review would not have had a long temporal archive to work with for satellites such as Aqua, Terra or Sentinel 1 and 2. Still, the most recent 5 years within the scope of our review had near-even proportions of cross-sectional and multi-temporal study design each year and on average (see table 5 of the supplementary file). Further multi-temporal analysis in EJ is important to consider as it can support characterization of cumulative impacts, which refers to the multiple environmental and social stressors communities face over time that can additively increase vulnerability and harm health [152, 153].

There were many instruments and datasets being used for EJ analyses (see table 3), but interestingly, little variation in type of satellite instrument (passive or active). Passive sensors, the most common type of satellite sensors, measure the reflected naturally emitted energy from the sun, whereas active sensors, which include SAR and light detection and ranging, emit their own source of light and measure the scattered light reflected to the sensor. Only one study used an active sensor, specifically SAR, in the measurement of a primary independent variable [110]. Active sensors offer expansive capabilities, including being able to see through clouds, and applications such as monitoring forests, floods, oil spills, landslides, and subsidence, that have intersections with EJ [154, 155]. The upcoming joint mission between NASA and the indian space research organization to launch a satellite carrying SAR instruments is well-poised to advance the use of SAR for EJ. We also found only two examples of commercial satellite imagery used [40, 44]. Commercial satellites can offer much higher spatial and temporal resolution than publicly available satellites, which could improve the granularity of EJ analyses. However, the financial cost of commercial satellite imagery may present barriers to EJ practitioners. These trends show there is an opportunity for more literature that broadens the use of satellite data for EJ topically, geographically, and temporally and in terms of imaging technique and source.

### 4.2. Strengths and limitations of satellite data for EJ and public health

This review demonstrates the value and potential of satellite data to advance the scientific understanding of inequitable exposures and impacts from environmental hazards. This paves the way for both researchers and community stakeholders to interrogate the root causes of inequities and assess policy interventions that may ameliorate those disparities. A key advantage frequently noted by the studies included in this review is the quasi-complete spatial coverage enabled by satellite data, which stands in contrast to the incomplete and uneven coverage of ground-based monitors. Ground-based monitors can be particularly sparse in rural areas, neighborhoods with more low-income households, and some areas occupied by certain minority populations. In such circumstances, satellite-derived data can improve accuracy in quantifying disparities in exposure and impact for those aforementioned populations [49, 62, 63, 76, 79, 82, 83, 95, 96, 137]. This spatial coverage also facilitates increased sample size for health effects studies and can reduce measurement error [76]. Studies using wide-coverage satellite data on air pollutants have also revealed important differences in health outcomes associated with PM_2.5_ exposure in urban versus rural areas [49, 76, 96]. Similarly, satellite-derived temperature data aid in understanding heat-related health burdens and mortality in rural communities with a dearth of ground temperature monitors [62, 63]. In the context of green space, wide-coverage satellite data have exposed the persistence of disparities in access to availability and accessibility of green space in census tracts nationally [17, 43–45, 51, 52, 54]. Remotely-sensed data are available at increasingly high spatial resolution, which supports estimates of access to environmental benefits (e.g. green space) or exposure to environmental hazards (e.g. air pollution, heat) at the census block group to census tract levels, and enables linkages to other small-area data on economic, environmental, and/or demographic characteristics (e.g. [40, 43, 46, 49, 90, 93, 137]).

Epidemiological studies especially benefit from increases in spatial resolution of exposure data, which can be more closely linked to high-resolution local health data [22, 42, 46, 116, 156]. For example, PM_2.5_ disparities have been found to be more pronounced [157] and PM_2.5_-attributable mortality estimates have been found to be higher [158] when using air pollutant data at finer spatial scales. Similarly, Huang *et al* [58], use census block group boundaries in Baltimore, Maryland to evaluate communities that are socially vulnerable in small-area geographies that are disproportionately vulnerable to high LST and may be at increased risk for heat-related mortality. The ability to link satellite data with health data could also improve public health monitoring and screening tools, such as the centers for disease control and prevention’s (CDC) EJ index [159] and national environmental health tracking network (tracking network) [160], which could benefit from increased timeliness of environmental quality data (e.g. green space, artificial light at night) that may lag when relying solely on modeled data. However, challenges can arise when aligning satellite data to be compatible with public health data, such as processing gridded data to match administrative units used in public health, like counties and census tracts. The tracking network addresses this gap by processing and hosting publicly available satellite data that align with geographic units that can be leveraged by public health researchers and practitioner [161].

Finally, the relatively rapid availability of direct satellite measurements has allowed researchers to quickly examine the EJ implications of policies or natural events, including the COVID-19 pandemic (e.g. [50, 53, 93, 98, 137]). In addition, the multitemporal advantage of satellite data has been leveraged to evaluate and track changes in the suitability of environmental indicators that are associated with environmental injustices over time [48] and evaluate persistence of disparities [19, 57, 59, 72, 80, 84, 94, 104].

There are important limitations associated with the use of satellite-derived data. A commonly cited limitation is the spatiotemporal gaps in coverage due to, for example, cloud cover, surface reflectance, height or presence of specific types of vegetation, and daytime light. Transforming direct satellite measurements to indirect measurements within physical or statistical modeling frameworks may address some of these spatiotemporal coverage gaps. In addition, SAR satellite data provides a robust alternative for some applications as the data can capture images at night and during cloudy and smoky scenes. Regarding greenspace usage, metrics such as the NDVI are not able to fully distinguish between the quality of vegetation and may fail to capture vegetation that might detract from community wellbeing [48]. For example, conducting ground-level site visits to evaluate the quality and accessibility of green spaces identified using satellite imagery can provide a more comprehensive understanding of potential EJ disparities and health impact [162]. As for temperature usage, remotely-sensed measurements of surface skin temperature are limited in their usability at scales relevant to human health [163]. For example, they are not fully representative of exposure experienced at 1–2 meters in height [124], and measurements are oftentimes biased toward horizontal surfaces and are unable to illustrate canopy layer air temperature [125] factors that are important in characterizing environmental health. In the urban environment in particular, satellite-derived LST can be highly uncertain and is oftentimes overestimated [125, 164]. With air pollution usage, many relevant pollutants cannot be directly measured by satellite instruments due to interference from other constituents in Earth’s atmosphere [165, 166]. Since health impact assessments require near-surface concentrations of pollutants as inputs, the direct measurements, or atmospheric columnar levels, made by satellites cannot be input to these assessments. Additionally, satellite-measured air pollutants might not be highly correlated with surface-level pollutant concentrations, which makes direct measurements of these quantities less applicable to EJ research. These limitations suggest that direct satellite measurements alone may not be sufficient to draw EJ conclusions, but coupled with other monitoring or modeling methods to form indirect measurements can achieve estimated surface-level concentrations with a suitable resolution and coverage for health-focused EJ research.

Future research should explore satellite data limitations when designing and evaluating policies and programs targeting EJ. Existing studies, while not focused on EJ outcomes, suggest that biases and errors in satellite data are significant when quantified in socioeconomically meaningful terms. For example, Fowlie *et al* [139] argue that the error structure for satellite-based PM_2.5_ data products is poorly understood and highlight the importance of further exploring the limitations of these data. Other studies have examined the consequences of bias and error in satellite data for impact evaluation and causal inference in policy analysis [167, 168].

### 4.3. Implications for U.S. government agencies

The strengths and limitations of satellite data for EJ applications identified in our review also have direct implications for agencies that launch and operate EO satellites, regulate environmental quality, and address EJ. In the U.S., the most relevant agencies are NASA, national oceanic and atmospheric association, U.S. geological survey (USGS), CDC, and EPA, all of which have prioritized environmental and climate justice in response to a series of executive orders. Most recently, executive order 14 008 (tackling the climate crisis at home and abroad) requires agencies to develop ‘programs, policies, and activities to address the disproportionately high and adverse human health, environmental, climate-related and other cumulative impacts on communities. that are disadvantaged’ [169]. For example, NASA’s Earth science division has established specific goals for its equity and EJ strategy, held community listening sessions, and issued two solicitations for proposals to advance progress on equity and EJ through uses of Earth science, geospatial, and socioeconomic information [170]. As NASA is launching new application-driven Earth-observing satellite missions (e.g. TEMPO, PACE, MAIA), the utility of satellite data for environmental health and EJ applications is expected to expand dramatically in the coming years. These efforts can build upon the methodologies summarized in our review and may be able to address some existing gaps, such as greater leveraging of the temporal dimension of satellite data. We also note that the EPA’s online screening tool, EJScreen, and the CEJST developed by the Biden administration could further leverage satellite-derived data, similar to other screening tools such as CalEnviroScreen and the CDC tracking program’s EJ dashboard. For example, the CDC tracking program’s EJ dashboard currently incorporates satellite data products (e.g. forecasted air quality data from GEOS-composition forecasting system [171] concurrently with health and other sociodemographic data (https://ephtracking.cdc.gov/Applications/ejdashboard/).

In addition to initiatives in U.S. federal agencies, methodologies summarized in our review may inform EJ applications in state and local government agencies. Many of these agencies across the country have developed EJ offices and agendas that may benefit from the use of satellite data, particularly when local data collection and analysis capabilities are limited. As additional motivation, the Justice40 initiative within executive Order 14 008—which has a goal of directing 40% of overall benefits of certain federal investments to communities that are marginalized, underserved, and overburdened by pollution—involves transfer of funds from federal agencies to state and local agencies that have some degree of discretion over eventual investment in disadvantaged communities [172]. With our reviews’ findings that higher spatial resolution data can yield more refined understandings of the distribution of environmental burdens, we suggest more agency-level adoption of satellite data as an input into screening tools for more accurate and timely detection of communities affected by environmental hazards.

### 4.4. Implications for policymakers, planners, and communities

Our findings also have implications for the use of satellite data to inform public policies, land-use planning, and other interventions that seek to influence EJ outcomes. While many of the studies we reviewed conclude with recommendations that satellite data be incorporated into the design and implementation of social, health, and environmental policies, only a handful of these studies identify specific policy contexts in which such adoption might take place. Several studies [45, 64] show that analysis using satellite data yields policy recommendations that are different from what would result from analysis of ground-based data alone, but the authors do not examine whether such use of satellite data in policy is administratively, politically, or socially feasible. A closer alignment between development of satellite-based decision support tools and actual policy impact is likely to require progress along two dimensions. First, greater policy and community engagement on the part of Earth scientists and remote sensing experts will help ensure that satellite data products can influence decisions that affect EJ outcomes [173]. Second, policymakers and urban planners can pro-actively seek opportunities to adopt satellite data to improve decisions and EJ outcomes. Such adoption may require costly modifications in agency protocols or guidance, and possibly legislative action. For example, while several of the studies we reviewed on air pollution measurement advocate for the increased use of satellite-derived data to inform EJ policy and planning, in many cases, legislation explicitly requires monitoring at ground level, which excludes satellite data as a source of information to drive decisions such as the determination of attainment with national ambient air quality standards [9]. One example of successful adoption in policy is that EPA guidance now allows states to use satellite data to identify exceptional events (e.g. fireworks, prescribed fires) that have a one-time impact on air quality, and requests that those measurements be excluded from national ambient air quality standards attainment and design value calculations [174, 175].

Beyond the policy realm, the literature overwhelmingly showed a gap in research conducted in partnership with community organizations, such as social justice, environmental, or urban planning nonprofits and coalitions, that could use satellite data to inform decision-making for local interventions to mitigate EJ inequities. This may be due to authors simply not reporting on their community engagement in scientific articles. Several NASA programs facilitate researcher engagement with community groups to address environmental, public health, and public policy issues (e.g. urban heat, disasters, air quality) through interdisciplinary research projects that apply NASA EO (e.g. DEVELOP, health and air quality applied sciences team, SERVIR). These programs have enabled several studies and collaborations with organizations to apply satellite data for EJ [176]. Advancing participatory research approaches can not only yield more rigorous and accurate science, but also ensure appropriately designed, targeted and executed interventions that serve a community’s best interests [177]. Remote sensing scientists and epidemiological researchers employing satellite data for EJ should look to best practices from scholarship in community based participatory action research (CBPAR/CBPR) [178] on how to conduct collaborative research for greater impact towards EJ.

## 5. Conclusion

In this scoping review, we synthesized evidence that used satellite data, in combination with other information, to evaluate environmental injustice in the U.S. The review showed that the use of satellite-derived data further cements findings that communities that are racially and ethnically marginalized, people with lower SES, and other populations that have been minoritized are disproportionately exposed to environmental risk factors and have less access to environmental benefits. These disparities in exposure and access were often associated with adverse health outcomes, such as increased cardiovascular and respiratory diseases, mental illness, hospitalizations, and mortality. The review also identified trends in the application of satellite data and highlighted how certain qualities of satellite-derived data enables assessment of disparities at scales and precision not previously possible. Research using satellite data for EJ can also contribute to efforts to mitigate inequities such as through supporting targeted interventions or planning and policy changes, but significant work remains to facilitate the application of satellite data for policy impact. Future research efforts should apply satellite data to address understudied EJ-relevant environmental categories, populations, and geographies, leverage the spatiotemporal resolution of satellite data for multitemporal studies, and design studies in collaboration with affected communities to conduct the most relevant science and identify effective, community-centered interventions.

## Supplementary Material

Supplementary material 2

Supplementary material 1

## Figures and Tables

**Table 1.** Main and expanded terms in literature search.

Main terms	Expanded terms
Satellite data	Satellite, earth observation, remote sensing, satellite remote sensing
Environmental justice	Environmental injustice, environmental equality, environmental inequality, environmental equity, environmental inequity
Environmental health	Health disparities, public health, environmental epidemiology
Demographic	Race, racial, ethnic, ethnicity, minorities, nationality, income, socioeconomic, socioeconomic status
Heat	Temperature, extreme heat, extreme temperature, heat risk, urban heat, thermal equity, thermal inequity, urban heat index
Green space	Urban green space, green space, parks, trees, vegetation
Pollution	Air pollution, air quality, light pollution, noise pollution, water pollution, water quality
Floods	Flood, flooding, flood inundation
Disparities	Health disparities, inequality, inequity

**Table 2.** Studies included in our review that explored green space (A), temperature (B), air pollution (C), and other environmental hazards (D) as the primary environmental category of interest. For each study, the geographic scope, study location, and social variables analyzed are briefly summarized.

A				
No.	Reference	Geographic scope	Location	Social variable(s)
1	Landry and Chakraborty [40]	City	Tampa, Florida	Race, ethnicity, income, housing tenure
2	Jesdale *et al* [17]	National	U.S.	Race, ethnicity, residential segregation
3	Zhou and Kim [41]	Multi-city	Illinois	Race, ethnicity
4	Gronlund *et al* [42]	Multi-city	Michigan	Age, race, sex, educational level, marital status
5	Saporito and Casey [43]	National	U.S.	Race, poverty
6	Schwarz *et al* [44]	Multi-city	U.S.	Race, ethnicity, income, education, housing age
7	Casey *et al* [45]	Country	U.S.	Race, ethnicity
8	Brown *et al* [46]	County	Miami-dade county, Florida	Income
9	Lara–Valencia and Garcia–Perez [47]	City	Phoenix, Arizona	Ethnicity
10	Schwarz *et al* [48]	City	Toledo, Ohio	Housing vacancy, race, wealth, education
11	Saporito and Casey [43]	County	U.S.	Age, sex
12	Son *et al* [49]	State	North Carolina	Race, ethnicity, age, gender, education, marital status
13	Kondo *et al* [50]	City	Philadelphia, Pennsylvania	Race, education, age, employment, poverty, income, housing vacancy
14	Fong *et al* [51]	Country	U.S.	Immigrant status, region of origin
15	Namin *et al* [52]	Multi-city	U.S.	Redlining
16	Lu *et al* [53]	Multi-city	U.S.	Race
17	Nardone *et al* [54]	Multi-city	U.S.	Redlining, race, median home value, employment, high school diploma, homes with a radio, number of homes needing major repairs, number of people per housing unit
B				
No.	Reference	Geographic scope	Location	Social variable(s)
1	Harlon *et al* [55]	City	Phoenix, Arizona	Ethnicity, income
2	Buyantuyev and Wu [56]	City	Phoenix, Arizona	Income, housing (number of households, number of housing units, housing age)
3	Jenerette *et al* [57]	City	Phoenix, Arizona	Income
4	Huang *et al* [58]	City	Baltimore, Maryland	Race, ethnicity, age, education, income, poverty, crime
5	Chow *et al* [59]	City	Phoenix, Arizona	Income, ethnicity, age, mobility, nativity
6	Harlan *et al* [60]	County	Maricopa county, Arizona	Race, ethnicity, age, poverty, education
7	Mitchell and Chakraborty [61]	County	Pinellas county, Florida	Race, ethnicity, SES (poverty, homeownership)
8	Lee *et al* [62]	Region	Southeastern U.S.	Age, sex, race, education, urbanicity
9	Shi *et al* [63]	Region	Southeastern U.S.	Age
10	Pearsall [64]	City	Philadelphia, Pennsylvania	Poverty, income, race, employment
11	Mitchell and Chakraborty [61]	Multi-city	U.S.	Race, ethnicity, SES (income, homeownership, education), segregation
12	Sanchez and Reames [65]	City	Detroit, Michigan	Income, race, ethnicity
13	Chakraborty *et al* [66]	National	U.S.	Income, race
14	Hoffman *et al* [67]	Multi-city	U.S.	Redlining
15	Wilson [68]	Multi-city	Baltimore, MD; Dallas, TX; Kansas city, MO	Redlining
16	Dialesandro *et al* [69]	Multi-city	Southwestern U.S.	Income, race, ethnicity
17	Carrion *et al* [70]	Region	Northeastern U.S.	Social vulnerability index (SVI; based on 15 census variables within the domains of socioeconomic status, household composition and disability, minority status and language, and housing and transportation)
18	Hsu *et al* [71]	National	U.S.	Income, race, ethnicity
19	Benz and Burney [72]	Country	U.S.	Income, race, ethnicity, elderly, citizenship, single parenthood, education
20	Rivera *et al* [73]	County	Santa Clara, California	Income, race, ethnicity, education, employment, housing (housing value, rent value), homeownership, households with vehicle
21	Muse *et al* [74]	County	Fulton county, Georgia	Income, race, age, education
22	Manware *et al* [75]	Country	U.S.	Redlining, race, ethnicity, age, employment, poverty, disability, language, housing (occupied houses built before 1980), nativity
C				
No.	Reference	Geographic scope	Location	Social variable(s)
1	Kloog *et al* [76]	State	Massachusetts	Education
2	Clark *et al* [77]	Country	U.S.	Race, ethnicity, income, age, education, urbanicity
3	Voorheis [78]	Country	U.S.	Income
4	Clark *et al* [19]	Country	U.S.	Race, ethnicity, income, age, education, urbanicity
5	Di *et al* [79]	Country	U.S.	Race, ethnicity, Medicaid eligibility, sex
6	Rosofsky *et al* [80]	State	Massachusetts	Race, ethnicity, income, education, urbanicity
7	Sullivan and Krupnick [9]	Country	U.S.	Race, ethnicity, education, income
8	Awad *et al* [81]	Country	U.S.	Race
9	Chang *et al* [82]	City	Jackson, MS	Race, sex
10	Lee [83]	State	California	Race, ethnicity, education, poverty
11	Colmer *et al* [84]	Country	U.S.	Race, ethnicity, unemployment, poverty, education, occupation
12	Currie *et al* [85]	Country	U.S.	Race, ethnicity
13	Demetillo *et al* [86]	City	Houston, Texas	Race, ethnicity, income
14	Qiu *et al* [87]	State	Massachusetts	Neonatal sex, maternal age, race, education
15	Bevan *et al* [88]	Country	U.S.	Social deprivation index (SDI; based on poverty, employment, renter status, housing conditions, education, car ownership)
16	Castillo *et al* [89]	City	Washington, DC	Education, employment, income, race, ethnicity, life expectancy at birth
17	Demetillo *et al* [90]	Multi-city	U.S.	Race, ethnicity, income
18	deSouza *et al* [10]	Country	U.S.	Medicaid eligibility, age, sex, race, ethnicity
19	Fong and Bell [91]	Country	U.S.	Immigrant status, region of origin, time since immigration
20	Khanum *et al* [92]	County	San Diego, CA	Environmental justice communities defined by CalEnviroScreen
21	Kerr *et al* [93]	Country	U.S.	Race, ethnicity, income, education, vehicle ownership
22	Liu *et al* [94]	Country	U.S.	Race, ethnicity, income, urbanicity
23	Qian *et al* [95]	Region	Southeastern U.S.	Sex, race, age, Medicaid eligibility, urbanicity, area-level SES indicator
24	Son *et al* [96]	State	North Carolina, Michigan	Urbanicity, income
25	Yazdi *et al* [97]	Country	U.S.	Age, sex, race, Medicaid eligibility, household income, population density
26	Zhang *et al* [30]	State	New York	Race
27	Bluhm *et al* [98]	State	California	Race, ethnicity
28	Boing *et al* [99]	Country	U.S.	Income, poverty, population density, race, education
29	Chakraborty *et al* [100]	Country	U.S.	Race, ethnicity, age, sex, disability status, income, urbanicity
30	Dressel *et al* [101]	Census Tract	New York city-Newark	Race, ethnicity, poverty
31	Heft–Neal *et al* [102]	State	California	Race, ethnicity, income
32	Hrycyna *et al* [103]	Multi-city	U.S.	Redlining
33	Jbaily *et al* [104]	Country	U.S.	Race, ethnicity, income
34	Lane *et al* [105]	Multi-city	U.S.	Redlining, race, ethnicity
35	Lee and Lee [106]	Country	U.S.	Income, race, age
36	Nowell *et al* [107]	Region	South Florida	Race, income
37	Terrell and James [108]	State	Louisiana	Race, age, employment, poverty
38	Wei *et al* [109]	Country	U.S.	Area deprivation index (ADI; based on education, employment, housing quality, poverty)
D				
No.	Reference	Geographic scope	Location	Social variables
1	Guidry and Margolis [110]	State	North Carolina	Race, income
12	Hendryx [111]	Region	Appalachia	Poverty, education, race, metropolitan area
3	Johnston *et al* [112]	Region	South Texas	Race, ethnicity
4	Nadybal *et al* [113]	Country	U.S.	Race, ethnicity, renter status, income

**Table 3.** Satellite instruments and satellite-derived data products used in ⩾ 3 of the included studies.

Environmental justice topic	Environmental variable	Tools Spatial coverage Spatial resolution Temporal coverage Temporal resolution	Data access	Literature using datasets included in review
Air pollution	Nitrogen dioxide (NO_2_)	**TROPOMI** Global 3.5 km × 5.2 km 2018-present Daily	www.tropomi.eu/data-products/nitrogen-dioxide	Demetillo *et al* [86]; Demetillo *et al* [90]; Kerr *et al* [93]; Bluhm *et al* [98]; Hrycyna *et al* [103]; Dressel *et al* [101]
		**Di *et al*** [117] **NO_2_** Continental United States 1 km × 1 km 2000–2016 Daily	https://sedac.ciesin.columbia.edu/data/set/aqdh-no2-concentrations-contiguous-us-1-km-2000-2016	Yazdi *et al* [97], Qian *et al* [95]; Wei *et al* [109]
	Particulate matter 2.5 *μ*ms or less in diameter (PM_2.5_)	**VanDonkelaar *et al*** [118] **surface PM**_**2.5**_ Global 0.01° × 0.01° (**~** 1 km × 1 km) 1998–2021 Monthly, annual	https://sites.wustl.edu/acag/datasets/surface-pm2-5/	Castillo *et al* [89]; Sullivan and Krupnick [9]; Terrell and James [108]; Bevan *et al* [88]; Fong *et al* [91]; Nowell *et al* [107]; Boing *et al* [99]
	Ozone (O_3_)	**Di *et al*** [119] **PM**_**2.5**_ Contiguous U.S. 1 km × 1 km 2000–2016 Daily, annual **Requia *et al*** [120] Contiguous U.S. 1 km × 1 km 2000–2016 Daily *Note that this dataset is an update of Di et al (2016), which estimated O*_*3*_ *from 2000–2012 for the same domain and spatial resolution*.	https://sedac.ciesin.columbia.edu/data/set/aqdh-pm2-5-concentrations-contiguous-us-1-km-https://sedac.ciesin.columbia.edu/data/set/aqdh-o3-concentrations-contiguous-us-1-km-2000-2016	Currie *et al* [85]; Di *et al* [79]; Awad *et al* [81]; Yazdi *et al* [97]; deSouza *et al* [10], Qiu *et al* [87]; Wei *et al* Di *et al* [79]*; Yazdi *et al* [97]^†^; Wei *et al* [109]^†^ * Using Di *et al* (2016) ^†^ Using Requia *et al* (2021)
	Multiple pollutants	**CACESLUR model for carbon monoxide (CO), sulfur dioxide (SO**_2_ **), particulate matter 10 *μ*ms or less in diameter (PM**_**10**_ **), O**_**3**_ **, NO**_**2**_ **, and PM**_**2.5**_ Contiguous U.S. National, state, county, census tract, census block group 1979–2015 Annual	www.caces.us/data	Liu *et al* [94]; Lane *et al* [105], Chakraborty *et al* [100]
Climate	Temperature	**Aqua MODIS land surface temperature and emissivity (MYD11a1)** Global 1 km 2002—Present Daily (daytime and nighttime)	https://lpdaac.usgs.gov/products/myd11a1v006/	Benz & Burney [72], Hsu *et al* [71], Chakraborty *et al* [66]
		**Landsat 5 thematic mapper** Global 120 m 1984–2013 16 d	https://earthexplorer.usgs.gov	Mitchell and Chakraborty [61]; Mitchell and Chakraborty [18]; Jenerette *et al* [57]
		**Landsat 7** Global 30 m 1999–2022 16 d	https://earthexplorer.usgs.gov	Jenerette *et al* [57]; Harlan [55]; Huang *et al* [58]; Chow, Chuang, & Gober [59]; Harlan *et al* [60] Pearsall [64]; Muse *et al* [74]; Dialesandro *et al* [69]; Rivera *et al* [73]; Sanchez & Reames [65]; Hoffman *et al* [67]; Wilson [68]
		**Landsat 8 thermal infrared sensor** Global 100 m 2013—Present 16 d	https://earthexplorer.usgs.gov	
Built environment	Green space	**Landsat 7** Global 30 m 1999–2022 16 d	https://earthexplorer.usgs.gov	Saporito & Casey [43]; Jenerette *et al* [57]; Harlan [60]
		**Landsat 8 operational land imager (OLI) and thermal infrared sensor (TIRS)** Global 30 m (**OLI**); 100 m (**TIRS**) 2013- present 16 d	www.arcgis.com/home/item.html?id=a1c373b16db34ef687ddae7c482e0b27 https://earthexplorer.usgs.gov/	Fong *et al* [51]; Kondo *et al* [50]; Lu *et al* [53]; Schwarz *et al* [48]
		**Terra modis** Global 250 m 1999—present 1–2 d (composite images every 16 d)	[link not given in paper but specific product (MOD13Q1) given] https://lpdaac.usgs.gov/products/mod13q1v006/ https://lpdaac.usgs.gov/products/mod13q1v061/	Casey *et al* [45]; Fong *et al* [51]; Heo & Bell [116]; Son *et al* [49]; Mitchell and Chakraborty [18]; Nardone *et al* [54]; Son *et al* [96]
		**National land cover datasets (NLCD)** Continental United States 30 m 1992-present 10 year repeat cycle prior to 2006; 5 year repeat cycle 2006-present	www.mrlc.gov/data	Gronlund *et al* [42]; Jesdale *et al* [17]; Lu *et al* [53]; Namin *et al* [52];

Satellite 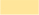

Satellite-derived or –incorporating 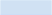

## Data Availability

All data that support the findings of this study are included within the article (and any supplementary files).

## Footnotes

13 A health disparity refers to a plausibly avoidable, systematic health difference adversely affecting a socially, economically, or environmentally disadvantaged group. This definition does not require establishing that the health difference was caused by social, economic, or environmental disadvantage. A health inequity is a particular kind of health disparity that is reasonably believed to reflect injustice. More information available at www.cdc.gov/healthcommunication/Health_Equity_Lens.html.

14 Annotations and initial drafts of the review paper were developed after Phase 1 by Author Sayyed.

15 Three studies that were not subject to peer-review were available as working papers or reports within our date range of 2000–2022. These were conducted by the Resources for the Future nonprofit research institution, the National Bureau of Economic Research, and the US Census Bureau Center for Administrative Records Research and Applications. These three studies were included as an exception to our inclusion criteria due to their unique contributions and relevance to addressing the research question of how satellite data can advance policy impact.

16 For the purposes of this review, ‘cross-sectional’ articles use satellite remote sensing data from a single point in time, or aggregate remote sensing data into a single time period for analysis. ‘Multi-temporal’ articles include analyses that incorporate repeated measures of remote sensing data and/or follow a unit of analysis over time. Example study designs that would be categorized as multi-temporal include cohort studies, time-series analyses, time-stratified case-crossover studies, or other longitudinal analyses.
