# Supplementary material for: Satellite data for environmental justice: a scoping review of the literature in the United States
Source: Environ Res Lett. Author manuscript; Available in PMC 2024 Oct 7. (PMC11457489; doi:10.1088/1748-9326/ad1fa4)
Supplement: Supplementary material 1 [file NIHMS2025626-supplement-Supplementary_material_1.docx]

Online supplementary materials.

**Satellite Data for Environmental Justice: A Scoping Review of the Literature in the United States**

Contents

**Supplementary Table 1.** Data Extraction Form for Team Members Reviewing each Study.

**Supplementary Table 2.** Included Articles (n = 81) with Extracted Attributes including Primary Exposure of Interest, Year of Publication, Study Location, Study Design, Geographic Scope, and Social Variables. *(uploaded as separate .xlsx file)*

**Supplementary Table 3.** All Satellite Instruments and Satellite-Derived Data Products used in the Studies Included in our Review.

**Supplementary Table 4.** Primary/downloadable Socioeconomic and Demographic Datasets used in the Studies Included in our Review.

**Supplementary Table 5.** Number of studies included in our review (n=81) by publication year and study design (i.e. cross-sectional or multi-temporal).

**Supplementary Table 1.** Data Extraction Form for Team Members Reviewing each Study.

| **#** | **Question** | **Answer Text** | **Answer Type** |
| --- | --- | --- | --- |
| Article Information | | | |
| 1 | Article Title | Full title of the article | Open response |
| 2 | Full Citation | Full citation of the article | Open response |
| 3 | Publication Year (online) | 2000, 2001, 2002, 2003, 2004, 2005, 2006, 2007, 2008, 2009, 2010, 2011, 2012, 2013, 2014, 2015, 2016, 2017, 2018, 2019, 2020, 2021, 2022 | Radio (one choice only) |
| Primary Social Categories | | | |
| 4 | Was ‘race’ used as an indicator over which injustice is examined? | Yes (Y); No (N) | Radio |
| 5 | Was ‘ethnicity’ used as an indicator over which injustice is examined? | Yes (Y); No (N) | Radio |
| Satellite Instrument Information | | | |
| 6 | Was a satellite instrument directly Used? | True; False | Radio |
| 7 | If a satellite instrument was directly used, what was the name of the satellite? | Name of the satellite instrument that was directly used in the study | Open Response |
| 8 | Was a satellite product used? | True; False | Radio |
| 9 | If a satellite-derived product was used, what was the name of the product? | Name of the satellite product used in the study | Open Response |
| 10 | What was the resolution of the extracted environmental data? | Resolution of extracted environmental data (i.e., 1km, 0.01 arc degrees, 250km, 30m, etc.) | Open Response |
| Study characterization | | | |
| 11 | What was the primary environmental topic evaluated in the study? | Air pollution; Green space; Temperature; Other Impacts | Radio |
| 12 | What was the secondary environmental topic evaluated in the study? | Air pollution; Green space; Temperature; Other Impacts | Radio |
| 13 | What variables were measured to characterize environmental exposure? | - Air pollution (AOD, NO2, PM2.5, NOx, O3, PM10, SO2, CO) - Green space (NDVI, UTC, EVI, TCC) - Temperature (LST, UHRI, Air temperature, SAVI, SUHI, NDBI, BSA) - Other Impacts (flared gas, floods, mountaintop mining (MTM) area, artificial light at night) - Additional (COVID infection, Impervious surface) | Select all that apply |
| Study characteristics | | | |
| 14 | What was the geographic scope of the study? | Geographic scope in which the study was conducted (i.e., state, city, multi-city, county, country, region) | Open Response |
| 15 | What was the study location? | Name of the state, city(s), region, county, or country in which the study took place. | Open Response |
| 16 | Was this an international study? | True; False | Radio |
| 17 | What was the design of the study’s observations? | Multi-temporal; Cross-sectional | Radio |
| 18 | What type of study was the data used to inform? | DE; DV; HIA; PEA | Select all that apply |
| 19 | What scale was the analysis conducted at? | The scale at which the analysis was conducted (i.e., zip code, census block, census tract, neighborhood, grid cell, county, individual) | Open Response |
| 20 | What were all the social categories over which injustice was examined? | Social categories over which injustice was examined (i.e.:   - Income-related Variables: Income, wealth, poverty, employment, Medicaid eligibility, household income, maternal education, educational attainment, high school diploma, Tract-level socioeconomic status, place of work - Housing and residential variables: % of renter-occupied housing units, residential location, public housing, housing tenure, segregation, urban vs non-urban, population mobility, Metropolitan area, urbanicity, ​​quartiles of population density (as a measure of urbanicity, median home value, number of homes needing a major repair, number of homes with a radio, number of people per housing unit; - Social/demographic variables: race, ethnicity, marital status, age, gender, sex, maternal race; - Health-related variables: obesity rates, smoking status, life expectancy at birth, place of death, Medicare; - Variables related to immigrant status: immigrant status, foreign born, immigrant proportion; - Combined variables, other variables, and indexes: Home Owners Lending Commission (HOLC) grading, vehicle ownership, social deprivation index (SDI), national area deprivation index (ADI), socioeconomic index variable (created by Kondo et al. 2020: )) | Open Response |

**Supplementary Table 3.** All Satellite Instruments and Satellite-Derived Data Products used in the Studies Included in our Review.

| **Environmental Justice Topic** | **Environmental variable** | **Tools**  • Spatial coverage  • Spatial resolution  • Temporal coverage  • Temporal resolution | **Data Access** | **Literature using datasets included in review** |
| --- | --- | --- | --- | --- |
| Air pollution | Nitrogen Dioxide (NO_2_) | **TROPOMI**  • Global  • 3.5 km x 5.2 km  • 2018-present  • Daily | <http://www.tropomi.eu/data-products/nitrogen-dioxide> | Demetillo et al., (2020); Demetillo et al., (2021); Kerr et al., (2021); Bluhm et al., 2022; Hrycyna et al., (2022); Dressel et al. (2022) |
|  |  | **Bechle et al. (2015) Land Use Regression (LUR)**  • Continental United States  • Census blocks, block groups, and tracts  • 2000-2010  • Monthly, Annual | <http://spatialmodel.com/concentrations/Bechle_LUR.html> | Clark et al. (2017) |
|  |  | **Novotny et al. (2011) LUR**  **•**Continental United States  • Census blocks  • 2006  • Annual | N/A | Clark et al. (2014) |
|  |  | **Di et al. (2020)**  **•**Continental United States  • 1 km x 1 km  • 2000-2016  • Daily | <https://sedac.ciesin.columbia.edu/data/set/aqdh-no2-concentrations-contiguous-us-1-km-2000-2016> | Yazdi et al. (2021), Qian et al. (2021); Wei et al. (2022) |
|  |  | **SP_GC product_v1.01**  • North America  • 10 km x 10 km  • 2005-2011  • Annual | <https://sites.wustl.edu/acag/datasets/surface-no2/> | Voorheis (2016) |
|  |  | **Lee and Koutrakis (2014)**  **•**New England region in the United States (Connecticut, Massachusetts, Rhode Island)  • 1 km x 1 km  • 2005-2010  • Daily | N/A | Rosofsky et al. (2018) |
|  | Particulate Matter 2.5 microns or less in diameter (PM_2.5_) | **MODIS Aerosol Optical Depth (AOD)**  • Global  • 10 km x 10 km  • 2000–present  • Daily | <https://ladsweb.modaps.eosdis.nasa.gov/missions-and-measurements/science-domain/aerosol/> | Zhang et al. (2021); |
|  |  | **Van Donkelaar et al. (2021) Surface PM2.5**  • Global  • 0.01° × 0.01° (~ 1 km x 1 km)  • 1998–2021  • Monthly, Annual | <https://sites.wustl.edu/acag/datasets/surface-pm2-5/> | Castillo et al. (2021); Sullivan & Krupnick (2018); Terrell & James (2020); Bevan et al. (2021); Fong et al. (2021); Nowell et al. (2022); Boing et al. (2022) |
|  |  | **Di et al. (2016, 2019) PM2.5**  • Contiguous U.S.  • 1 km x 1 km  • 2000–2016  • Daily, Annual | <https://sedac.ciesin.columbia.edu/data/set/aqdh-pm2-5-concentrations-contiguous-us-1-km-2000-2016> | Currie et al. (2020); Di et al. (2017); Awad et al. (2019); Yazdi et al. (2021); deSouza et al. (2021), Qiu et al. (2020); Currie et al. (2023); Wei et al. (2022); Son et al. (2021) |
|  |  | **CalEnviroScreen PM2.5**  • California  • Census tract  • 2012–2014  • Annual (average of quarterly means) | <https://oehha.ca.gov/calenviroscreen/report/calenviroscreen-30> | Khanum et al. (2021; 10.1080/10962247.2021.1994053) |
|  |  | **Meng et al. (2019) PM2.5**  • North America  •  0.1° × 0.1° (~ 11.1 km x 11.1 km)  • 1981–2016  • Annual | <https://sites.wustl.edu/junmeng/dataset/historical-pm2-5-estimates-across-north-america/> | Colmer et al. (2020); Jbaily et al. (2022) |
|  |  | **Lary et al. (2015) PM2.5**  • Global  • 10 km x 10 km  • 1997-2014  • Daily (24-hour average) | N/A | Chang et al. (2019) |
|  |  | **Lee (2019) PM2.5** [https://doi.org/10.1021/acs.est.9b03799]  • California  • 1 km x 1 km  • 2016  • Annual average | N/A; census tract-scale PM2.5 estimates derived in part from Lee (2019) model available through CalEnviroScreen 4.0 at: https://oehha.ca.gov/calenviroscreen/report/calenviroscreen-40 | Lee (2019) |
|  |  | **Lee & Lee (2022) PM2.5**  • Metropolitan statistical areas in the United States  • 0.8 km x 0.8 km  • 2014  • Annual average | N/A | Lee & Lee (2022) |
|  |  | **Kloog et al. (2011) PM2.5**  • New England region in the United States (Connecticut, Maine, Massachusetts, New Hampshire, Rhode Island and Vermont)  • 10 km x 10 km  • 2000-2008  • Daily | N/A | Kloog et al. (2013) |
|  |  | **Kloog et al. (2014) PM2.5**  • Northeastern United States (Connecticut, Maine, Massachusetts, New Hampshire, Rhode Island, Vermont, New York, New Jersey)  • 1 km x 1 km  • 2003-2011  • Daily | N/A | Rosofsky et al. (2018) |
|  |  | **Lee et al. (2015) PM2.5** [https://doi.org/10.1038%2Fjes.2015.41]  • North Carolina, South Carolina, Georgia, Tennessee, Alabama, Mississippi, and Florida  • 1 km x 1 km  • 2003 - 2011  • Daily | N/A | Lee et al. (2016) |
|  | Ozone (O_3_) | **Requia et al. (2021)**  • Contiguous U.S.  • 1 km x 1 km  • 2000-2016  • Daily  *Note that this dataset is an update of Di et al. (2016), which estimated O3 from 2000-2012 for the same domain and spatial resolution.* | <https://sedac.ciesin.columbia.edu/data/set/aqdh-o3-concentrations-contiguous-us-1-km-2000-2016> | Di et al. (2017)*; Yazdi et al. (2021)^†^; Wei et al. (2022)^†^  * Using Di et al. (2016)  ^†^ Using Requia et al. (2021) |
|  | Smoke (horizontal) | **NOAA Hazard Mapping System**  MODIS, GOES, VIIRS  • North America  • Hand-drawn polygons  • 2005– Present  • ~2x daily | <https://www.ospo.noaa.gov/Products/land/hms.html> | Heft-Neal et al. (2022) |
|  | Multiple pollutants | **CACES LUR model for Carbon Monoxide (CO), Sulfur Dioxide (SO_2_), Particulate Matter 10 microns or less in diameter (PM_10_), O_3_, NO_2_, and PM_2.5_**  • Contiguous U.S.  • national, state, county, census tract, census block group  • 1979–2015  • Annual | <https://www.caces.us/data> | Liu et al., (2021); Lane et al., (2022)**,** Chakraborty et al. (2022) |
| Climate | Temperature | **Landsat 5 Thematic Mapper**  • Global  • 120 m  • 1984–2013  • 16 days | <https://earthexplorer.usgs.gov> | Mitchell et al. (2018); Mitchell and Chakraborty (2014); Jenerette et al. (2011) |
|  |  | **Landsat 7**  • Global  • 30 m  • 1999-2022  • 16 days | <https://earthexplorer.usgs.gov> | Jenerette et al. (2011); Harlan (2007); Huang & Cadenasso (2011); Chow, Chuang, & Gober (2012); Harlan et al. (2013) |
|  |  | **Landsat 8 Thermal Infrared Sensor**  • Global  • 100 m  • 2013 - Present  • 16 days | <https://earthexplorer.usgs.gov> | Pearsall (2017); Muse et al. (2022); Dialesandro et al. (2021); Rivera et al. (2022); Sanchez & Reames (2019); Hoffman et al. (2020); Wilson (2020) |
|  |  | **MODIS Terra Land Surface Temperature and Emissivity (MOD11A1)**  • Global  • 1 km  • 2000 - Present  • Daily (daytime and nighttime) | <https://ladsweb.modaps.eosdis.nasa.gov/archive/allData/61/MOD11A1/>  <https://earthexplorer.usgs.gov> | Benz & Burney (2021), Hsu et al. (2021) |
|  |  | **MODIS Aqua Land Surface Temperature and Emissivity (MYD11a1)**  • Global  • 1 km  • 2002 - Present  • Daily (daytime and nighttime) | <https://lpdaac.usgs.gov/products/myd11a1v006/> | Benz & Burney (2021), Hsu et al. (2021), Chakraborty et al. (2020) |
|  |  | **MODIS Aqua Land Surface Temperature and Emissivity (MYD11A2)**  • Global  • 1 km  • 2002 - Present  • 8 days | <https://lpdaac.usgs.gov/products/myd11a2v006/> | Chakraborty et al. (2020) |
|  |  | **Terra ASTER**  • Global  • 15, 30m, 90m  • 1999-present  • 16 days | <https://search.earthdata.nasa.gov/portal/idn/search?fi=ASTER> | Buyantuyev & Wu (2010) |
|  |  | **Shi et al. (2016) near-surface air temperature,** [https://doi.org/10.1038/srep30161]  • North Carolina, South Carolina, Georgia, Tennessee, Alabama, Mississippi, and Florida  • 1 km  • 2000-2014  • Daily | N/A | Lee et al. (2016); Shi et al. (2016) |
|  |  | **Carrión et al. (2021) near-surface air temperature**  • Maine, New Hampshire, Vermont, Massachusetts, Rhode Island, Connecticut, New York, New Jersey, Delaware, Pennsylvania, Maryland, West Virginia, and Virginia, plus Washington, DC  • ~1 km  • 2003 - 2019  • hourly | N/A | Carrión et al. (2021) |
|  |  | **NASA Daymet (Daily Surface Weather Data; v4) 2-meter temperature**  • Continental North America  • 1 km  • 1980-2021  • Daily | <https://daac.ornl.gov/cgi-bin/dsviewer.pl?ds_id=2129> | Manware et al. (2022) |
|  | Fire | **Visible Infrared Imaging Spectroradiometer (VIIRS)**  • Global  • 375 m  • 2012 - Present  • ~2x daily | <https://www.earthdata.nasa.gov/learn/find-data/near-real-time/firms> | Johnston et al. (2020) |
| Built environment | Green Space | **Landsat 1 Multispectral Scanner System (MSS)**  • Global  • 80 m  • 1972–1978  • 18 days | <https://earthexplorer.usgs.gov> | Jenerette et al. (2011) |
|  |  | **Landsat 2**  • Global  • 60 m  • 1975-1983  • 18 days | <https://earthexplorer.usgs.gov> | Schwarz et al. (2018) |
|  |  | **Landsat 3 MSS**  • Global  • 80 m  • 1973–1983  • 18 days | <https://earthexplorer.usgs.gov> | Jenerette et al. (2011) |
|  |  | **Landsat 5**  • Global  • 30 m  • 1984-2013  • 16 days | <https://earthexplorer.usgs.gov> | Schwarz et al. (2018); Jenerette et al. (2011) |
|  |  | **Landsat 7**  • Global  • 30 m  • 1999-2022  • 16 days | <https://earthexplorer.usgs.gov> | Saporito & Casey (2015); Jenerette et al. (2011); Harlan (2007) |
|  |  | **Landsat 8 Operational Land Imager (OLI) and Thermal Infrared Sensor (TIRS)**  • Global  • 30 m (**OLI**); 100 m (**TIRS**)  • 2013- present  • 16 days | [https://www.arcgis.com/ home/item.html?id=a1c373b16db34ef687ddae7c482e0b27](https://www.arcgis.com/)  <https://earthexplorer.usgs.gov/> | Fong et al. (2020); Kondo et al. (2020); Lu et al. (2021); Schwarz et al. (2018); Rivera et al. (2022); Wilson (2020) |
|  |  | **MODIS Terra**  • Global  • 250 m  • 1999 - Present  • 1-2 days (composite images every 16 days) | [link not given in paper but specific product (MOD13Q1) given]  <https://lpdaac.usgs.gov/products/mod13q1v006/>  <https://lpdaac.usgs.gov/products/mod13q1v061/> | Casey et al. (2017); Fong et al (2020); Heo & Bell (2019); Son et al. (2020); Mitchell and Chakraborty (2014); Nardone et al (2021); Son et al. (2021) |
|  |  | **MODIS Aqua Surface Reflectance (MYD09A1)**  • Global  • 500 m  • 2002 - Present  • 8 days | https://lpdaac.usgs.gov/products/myd09a1v006/ | Chakraborty et al. (2020) |
|  |  | **MODIS Terra Vegetation Indices (MOD13A2)**  • Global  • 1 km  • 2000 - Present  • 16 days | <https://lpdaac.usgs.gov/products/mod13a2v006/> | Benz & Burney (2021) |
|  |  | **MODIS Aqua Vegetation Indices (MYD13A2)**  • Global  • 1 km  • 2000 - Present  • 16 days | https://lpdaac.usgs.gov/products/myd13a2v006/ | Benz & Burney (2021) |
|  |  | **IKONOS**  •  Global  • 1 m  • 1999 - 2015  • 3 days | <https://catalog.data.gov/dataset/ikonos-2> | Landry & Chakraborty (2009) |
|  |  | **QuickBird Satellite Imagery**  • Global  • 0.6 m  • 2001-2015  • 1-3.5 days | N/A | Schwarz et al. (2015) |
|  |  | **Terra ASTER**  • Global  • 15 m  • 1999-present  • 16 days | <https://search.earthdata.nasa.gov/portal/idn/search?fi=ASTER> | Brown et al. (2018) |
|  |  | **National Agricultural Imagery Program (NAIP)**  • Continental United States  • 1 m  • 2003-present  • 3 year repeat cycle | [United States Department of Agriculture’s (USDA) Geospatial Data](https://datagateway.nrcs.usda.gov/GDGHome_DirectDownLoad.aspx)  [Gateway](https://datagateway.nrcs.usda.gov/GDGHome_DirectDownLoad.aspx) | Schwarz et al. (2015); Zhou & Kim (2013) |
|  |  | **National Landcover Datasets (NCLD)**  • Continental United States  • 30 m  • 1992-present  • 10-year repeat cycle prior to 2006; 5-year repeat cycle 2006-present | <https://www.mrlc.gov/data> | Gronlund et al. (2014); Jesdale et al. (2013); Lu et al. (2021); Namin et al. (2020); Hoffman et al. (2020); Pearsall (2017); Benz & Burney (2021); Chakraborty et al. (2020) |
|  |  | **Google Earth Pro**  • Global  • 30 m  • 1984 - present  • 16 days (via Landsat) | [did not link directly to source/data access]   <https://earth.google.com/web/@33.6054149,-112.125051,388.8836482a,154893.72247639d,35y,0h,0t,0r/data=CkoaSBJCCiUweDg3MmIxMmVkNTBhMTc5Y2I6MHg4YzY5YzdmODM1NGExYmFjGT5jumtkuUBAIcmL8Qa9BFzAKgdQaG9lbml4GAIgAQ> | Lara-Valencia & Garcia-Perez (2018) |
|  |  | **European Space Agency Climate Change Initiative (ESA CCI)**  **Land Cover**  • Global  • 300 m  • 1992 to 2015  • Annual | https://www.esa-landcover-cci.org/?q=node/164 | [Bontemps et al., 2013](https://www.sciencedirect.com/science/article/pii/S0924271620302082#b0020), Hsu et al. (2021) |
|  | Artificial nighttime light | **VIIRS/The New World Atlas of Artificial Sky Brightness**  • Global  • 30 arc-seconds (~1 km)  • 2012- ongoing  • Monthly | <https://datapub.gfz-potsdam.de/download/10.5880.GFZ.1.4.2016.001/> | Nadybal et al., 2020 |

Satellite ______

Satellite-derived or -incorporating ________

**Supplementary Table 4.** Primary/downloadable Socioeconomic and Demographic Datasets used in the Studies Included in our Review.

| **No.** | **Dataset** | **Description** | **Articles used in** |
| --- | --- | --- | --- |
| 1 | American Community Survey (ACS) 5-Year Estimates | (1) Population statistics, such as age, education, count for and self-reported race/ethnicity counts | Bluhm et al. (2022), Castillo et al. (2021), Chakraborty et al. (2022), Colmer et al. (2020), Kerr et al. (2021), Kondo et al. (2020), Rosofsky et al. (2018), Sullivan and Krupnick (2018), Terrell and James (2022), Mitchell & Chakraborty (2014), Pearsall (2017), Mitchell & Chakraborty (2018), Schwarz et al. (2018), Wilson (2020), Dialesandro et al. (2021), Saporito & Casey (2015), Gronlund et al. (2015), Hsu et al. (2021), Benz & Burney (2021), Rivera et al. (2022), Muse et al. (2022), Manware et al. (2022), Sanchez & Reames (2019), Chakraborty et al. (2020), Currie et al. (2020), Boing et al. (2022), Dressel et al. (2022), Jbaily et al. (2022), Nowell et al. (2022) |
|  |  | (2) Household income statistics | Demetillo et al. (2020), Mitchell & Chakraborty (2014), Pearsall (2017), Mitchell & Chakraborty (2018), Wilson (2020), Dialesandro et al. (2021), Saporito & Casey (2015), Gronlund et al. (2015), Heo & Bell (2019), Hsu et al. (2021), Benz & Burney (2021), Rivera et al. (2022), Muse et al. (2022), Sanchez & Reames (2019), Chakraborty et al. (2020), Clark et al. (2017), Awad et al. (2019), Heft-Neal et al. (2022), Jbaily et al. (2022), Nadybal et al. (2020) |
| 2 | ACS 1-Year Estimates |  | Voorheis et al. (2016) |
| 3 | Decennial U.S. Census (U.S. Census Bureau Database) | (1) Population statistics, such as age, education, and race/ethnicity counts | Casey et al. (2017), Colmer et al. (2020), Demetillo et al. (2020), Hrycyna et al. (2022), Kloog et al. (2013), Lane et al. (2022), Lara-Valencia & Garcia-Perez (2018), Rosofsky et al. (2018), Schwarz et al. (2018), Sullivan and Krupnick (2018), Harlon et al.(2007), Buyantuyev and Wu (2010), Jenerette et al. (2011), Huang et al. (2011), Chow et al. (2012), Harlan et al. (2013), Mitchell & Chakraborty (2018), Landry & Chakraborty (2009), Lu et al. (2021), Fong et al. (2020), Schwarz et al. (2015), Zhou & Kim (2013), Gronlund et al. (2015), Son et al. (2020), Jesdale et al. (2013), Heo & Bell (2019), Clark et al. (2014), Clark et al. (2017), Awad et al. (2019), Lee (2019), Currie et al. (2020), Fong & Bell (2021), Boing et al. (2022), Jbaily et al. (2022), Johnston et al. (2020), Nadybal et a. (2020) |
|  |  | (2) Household income statistics | Brown et al. (2018), Demetillo et al. (2021), Harlon et al. (2007), Buyantuyev and Wu (2010), Jenerette et al. (2011), Huang et al. (2011), Lee & Lee (2022), Chow et al. (2012), Harlan et al. (2013), Mitchell & Chakraborty (2018), Schwarz et al. (2018), Landry & Chakraborty (2009), Casey et al. (2017), Schwarz et al. (2015), Zhou & Kim (2013), Gronlund et al. (2015), Son et al. (2020), Jesdale et al. (2013), Heo & Bell (2019), Jbaily et al. (2022) |
|  |  | (3) Metropolitan and Micropolitan Statistical Areas and Components | Casey et al. (2017), Hrycyna et al. (2022) |
| 4 | Mapping Inequality (Nelson et al., 2021) | The University of Richmond’s Mapping Inequality project to identify the HOLC (Home Owners Loan Commission) codes. | Hrycyna et al. (2022), Lane et al. (2022), Hoffman et al. (2020), Wilson (2020), Namin et al. (2020), Nardone et al. (2021) |
| 5 | Income Inequality estimates (Voorheis, 2014) |  | Voorheis et al. (2016) |
| 6 | NASA's Socioeconomic Data and Applications Center's (SEDAC) Gridded Population of the World (GPW), Version 4 | Population estimates (total population and population by age and gender) by the Center for International Earth Science Information Network (CIESIN) at 30 Arc-Second (∼1 × 1 km) resolution | Castillo et al. (2021) |
| 7 | U.S. Centers for Medicare and Medicaid Services’ (CMS’) Master Beneficiary Summary File (MBSF) | Provides the age, gender, race/ethnicity, annual data on health outcomes | Brown et al. (2018), Shi et al. (2016), Di et al. (2017), Awad et al. (2019), deSouza et al. (2021), Qian et al. (2021), Son et al. (2021), Yazdi et al. (2021), Wei et al. (2022) |
| 8 | Rural–Urban Commuting Area (RUCA) codes | To account for urban–rural differences, codes developed by the US Department of Agriculture that classify census tracts using measures of population density, urbanization, and daily commuting | Chakraborty et al. (2022) |
| 9 | US Department of Housing and Urban Development (HUD) Picture of Subsidized Households | For each HUD-subsidized household, this database provides locational coordinates, number of occupied and unoccupied units, socio-demographic characteristics of resident households, and other relevant details. | Chakraborty et al. (2022) |
| 10 | ESRI Business Analyst dataset (ESRI 2017) | Annual population data at the block group level | Chang et al. (2019) |
| 11 | Comprehensive Housing Affordability Strategy Data | U.S. Housing and Urban Development’s Comprehensive Housing Affordability Strategy Data (2009–2013) (California Office of Environmental Health Hazard Assessment Citation 2017) | Khanum et al. (2021) |
| 12 | U.S Census Bureau Data for 1940 from the Individual Public Use Microdata Series National Historical Geographic Information Systems database | Census tract shapefiles and sociodemographic variables | Nardone et al. (2021) |
| 13 | Annual Social and Economic Supplement (ASEC) of the Current Population Survey (CPS) | Estimates of poverty levels, rates, and widely used measures of income | Heo & Bell (2019) |
| 14 | U.S. CDC’s Social Vulnerability Index | Social vulnerability ranking using 16 social factors (categorized by household characteristics, race/ethnic minority status, socioeconomic status, and housing type/transportation) derived from U.S. Census data. | Carrión et al. (2021) |
| 15 | Social Deprivation Index (SDI) | SDI is a composite measure of area level deprivation based on seven demographic characteristics collected in the American Community Survey and used to quantify the socio-economic variation in health outcomes. | Bevan et al. (2021) |
| 16 | Area Deprivation Index (ADI) | The Area Deprivation Index (ADI) allows for rankings of neighborhoods by socioeconomic disadvantage in a region of interest (e.g. at the state or national level). It includes factors for the theoretical domains of income, education, employment, and housing quality. | Wei et al. (2022) |
| 17 | White House’s Climate and Economic Justice Screening Tool | Disadvantaged community data | Manware et al. (2022) |
| 18 | Historic Redlining Scores for 2010 and 2020 US Census Tracts (Meier & Mitchell, 2022) | Historic redlining scores | Manware et al. (2022) |
| 19 | IPUMS National Historic Geographic Information System (NHGIS) (Manson et al. 2019) | Decennial census data providing population estimates by race, ethnicity and household income | Liu et al. (2021) |

**Supplementary Table 5.** Number of studies included in our review (n=81) by publication year and study design (i.e. cross-sectional or multi-temporal).

| *Year of Publication* | Percentage Cross-sectional studies each year | Percentage Multi-temporal studies each year |
| --- | --- | --- |
| 2005 | 100.00% | 0.00% |
| 2007 | 100.00% | 0.00% |
| 2009 | 100.00% | 0.00% |
| 2010 | 50.00% | 50.00% |
| 2011 | 50.00% | 50.00% |
| 2012 | 0.00% | 100.00% |
| 2013 | 50.00% | 50.00% |
| 2014 | 100.00% | 0.00% |
| 2015 | 66.67% | 33.33% |
| 2016 | 0.00% | 100.00% |
| 2017 | 25.00% | 75.00% |
| 2018 | 66.67% | 33.33% |
| 2019 | 40.00% | 60.00% |
| 2020 | 46.15% | 53.85% |
| 2021 | 38.89% | 61.11% |
| 2022 | 41.67% | 58.33% |
